# The *Arabidopsis* Domain of Unknown Function 1218 (DUF1218) Containing Proteins, MODIFYING WALL LIGNIN-1 and 2 (At1g31720/MWL-1 and At4g19370/MWL-2) Function Redundantly to Alter Secondary Cell Wall Lignin Content

**DOI:** 10.1371/journal.pone.0150254

**Published:** 2016-03-01

**Authors:** Ritesh Mewalal, Eshchar Mizrachi, Berdine Coetzee, Shawn D. Mansfield, Alexander A. Myburg

**Affiliations:** 1 Department of Genetics, Forestry and Agricultural Biotechnology Institute (FABI), University of Pretoria, Private bag X20, Pretoria, 0028, South Africa; 2 Department of Chemical Engineering, University of Pretoria, Private bag X20, Pretoria, 0028, South Africa; 3 Sappi Southern Africa, P.O Box 12796, Pretoria, 0087, South Africa; 4 Department of Wood Science, Faculty of Forestry, University of British Columbia, Forest Sciences Centre, 4030–2424 Main Mall, Vancouver, BC, V6T 1Z4, Canada; Iowa State University, UNITED STATES

## Abstract

DUF1218 is a land plant-specific innovation and has previously been shown to be associated with cell wall biology, vasculature patterning and abiotic/biotic stress response. The *Arabidopsis* genome encodes 15 members, two of which (At1g31720 and At4g27435) are preferentially expressed in the secondary cell wall depositing inflorescence stems. To further our understanding of the roles of DUF1218-containing proteins in secondary cell wall biology, we functionally characterized At1g31720 (herein referred to as *MODIFYING WALL LIGNIN-1* or *MWL-1*). Since related gene family members may contribute to functional redundancy, we also characterized At4g19370 (*MWL-2*), the most closely related gene to *MWL-1* in the protein family. Subcellular localization revealed that both *Arabidopsis* proteins are targeted to the cell periphery. The single T-DNA knockout lines, *mwl*-1 and *mwl*-2, and independent overexpression lines showed no significant differences in plant growth or changes in total lignin content relative to wild-type (WT) control plants. However, the double homozygous mutant, *mwl*-1/*mwl*-2, had smaller rosettes with a significant decrease in rosette fresh weight and stem height relative to the WT control at four weeks and six weeks, respectively. Moreover, *mwl*-1/*mwl*-2 showed a significant reduction in total lignin content (by *ca*. 11% relative to WT) and an increase in syringyl/guaiacyl (S/G) monomer ratio relative to the control plants. Our study has identified two additional members of the DUF1218 family in *Arabidopsis* as novel contributors to secondary cell wall biology, specifically lignin biosynthesis, and these proteins appear to function redundantly.

## Introduction

The plant cell wall is involved in a variety of physiological functions including mechanical support, growth, a physical barrier to pathogens, signalling, intercellular communication, and environmental interaction [1,2]. Genes encoding the plant-specific domain of unknown function 1218 (DUF1218) family have been implicated in several aspects of cell wall biology [3,4] and shown to function in vasculature patterning [5] and response to abiotic and biotic stresses [6–8]. Independent studies [4,9] reported expression correlation between a DUF1218-encoding gene (At4g27435) and the secondary cell wall-related cellulose synthase (CesA) genes. The cellulose content of a loss-of-function mutant line for At4g27435 remained unchanged relative to the WT control plants [4,9], despite the altered cellulose and pectin profile inferred using Fourier Transform Infrared Spectroscopy [4]. Ubeda-Tomas *et al*. (2006) later showed that the At4g27435 knock-out had a reduction in total xylem, but could not detect the cell wall chemistry changes reported earlier by Persson *et al*. (2005). In addition to At4g27435, another DUF1218-containing protein, At1g31720 (referred to as *MODIFYING WALL LIGNIN-1* or *MWL*-1 in this paper) is also preferentially expressed in the inflorescent stems of *Arabidopsis*. *MWL*-1 is also co-expressed with the three secondary cell wall cellulose synthase genes and several xylan and lignin-associated genes [10]. This combined with the findings that these DUF1218-containing protein homologs were conserved between and preferentially expressed in xylem of angiosperms and gymnosperms (Pavy et al., 2008), emphasizes the importance of the role of DUF1218 proteins in xylogenesis and/or secondary cell wall formation.

In this study, we aimed to functionally characterize At1g31720 (*MWL-1*) highlighted as important in xylem biology. We report that single knockout and overexpression of *MWL*-1 and the closely related DUF1218 member, At4g19370 (*MWL*-2), does not alter plant morphology or cell wall lignin content, but the double knockout, *mwl*-1/*mwl*2, moderately yet significantly decreases plant growth and total lignin content with a concurrent increase in S/G lignin monomer ratio relative to WT control plants. Our study reinforces the potential role of DUF1218-containing proteins in lignin biosynthesis and suggests functional redundancy between *MWL*-1 and *MWL*-2.

## Materials and Methods

### Plant growth conditions

All *Arabidopsis* seeds were surface sterilized and sown on 0.5x Murashige and Skoog agar plates (pH 5.8, 1% agar). If applicable, selection was carried out with 25 μg/ml glufosinate-ammonium (Sigma-Aldrich), 7.5 mg/ml sodium salt of sulfadiazin (Sigma-Aldrich) or 20 μg/ml HyClone^™^ Hygromycin B (Fisher Scientific). Seeds were stratified for 48 hours at 4°C, thereafter transferred to ~22°C for one week under constant light. Seedlings were planted out in peat moss bags (Jiffy Products International AS, Norway) and placed in a controlled growth room under 16 hours photoperiod using an OSRAM L 58W/965 BIOLUX light source at ~22°C with 60% relative humidity for a further six weeks. Three independent paired trials were conducted for each line where mutant/transgenic replicates were grown alongside the wild-type control replicates to minimize variation. All mutant and transgenics were in the Columbia-0 (Col-0) ecotype.

### Isolation of T-DNA insertion lines

*Arabidopsis thaliana* T-DNA knockout lines for *MWL-2* (SAIL_1209_H12) and *MWL-1* (GABI-Kat: 932A01) were obtained from the Nottingham *Arabidopsis* Stock Centre and GABI-Kat, respectively. Positive lines were selected on either glufosinate-ammonium or sodium salt of sulfadiazine. Homozygosity of the T-DNA lines was confirmed via genomic PCR screening using a combination of the T-DNA left border oligonucleotides and gene-specific oligonucleotides (S1 Table). Furthermore, the single homozygous T-DNA lines were crossed and subsequently self-pollinated to generate a homozygous *mwl-1/mwl-2* double mutant line that was selected on a combination of glufosinate-ammonium and sodium salt of sulfadiazine, and further confirmed using the aforementioned genomic PCR screening approach. The disruption of the native gene via the T-DNA insertion for all mutant plants was also confirmed at the transcript level using gene-specific oligonucleotides that flanked the T-DNA insertion site (S1 Table, S3A and S4A Figs). Briefly, NucleoSpin^®^ RNA Plant (Macherey-Nagel) and Improm-II^™^ Reverse Transcriptase cDNA synthesis kit (Promega) was used to extract total RNA from stem tissue of six-week-old plants and synthesize first-strand cDNA from 1 μg DNAse I-treated RNA, respectively. The cDNA was subsequently used as a template for PCR analysis with the following parameters: 3 minutes at 95°C, followed by 30 cycles at 95°C for 30 seconds, 58°C for 30 seconds and 72°C for 20 seconds and a final cycle at 72°C for 5 minutes. Actin2 (At3g18780) served as an internal PCR control using primers listed in S1 Table.

### Generation of overexpression lines

Gene sequences corresponding to *MWL-1* and *MWL-2* were amplified from *Arabidopsis* Col-0 cDNA using gene-specific primers (S1 Table) and cloned into pCR^®^8/GW/TOPO^®^ vector (Invitrogen^™^) and thereafter sequenced. The genes were recombined into the gateway-compatible binary expression vector, pMDC32 [11], downstream of a double 35S cauliflower mosaic virus (CaMV) promoter. pMDC32-*MWL-1* and pMDC32-*MWL-2* were then transformed into *Agrobacterium tumefaciens* (strain LBA4404) which was used to transform *Arabidopsis* Col-0 plants using the floral dip method [12]. Transgenic plants were selected on hygromycin and confirmed via PCR using a 35S-specific primer and the reverse gene-specific primers (S1 Table). T3 plants were used for subsequent phenotypic studies and overexpression of the transgene was confirmed with semi-quantitative PCR using the gene-specific oligonucleotides (S1 Table) and the aforementioned conditions (except 22 PCR cycles) on stem tissue (S3B and S4B Figs).

### Subcellular localization

Genes encoding MWL-1 and MWL-2 were amplified from *Arabidopsis* Col-0 cDNA using gene-specific forward primers and modified reverse primers where the stop codon was removed (S1 Table). The PCR products were cloned into the pENTR^™^/D-TOPO^®^ vector (Invitrogen^™^), sequenced verified, and thereafter recombined into the transient expression vector pSAT-DEST-GFP-N1B (CD3-1654, TAIR) downstream a double 35S CaMV promoter and in-frame with a C-terminal Green Florescent Protein (GFP). Protoplast isolation and PEG-calcium transfection was carried out in *Populus alba* × *tremula*, P717 protoplasts using the method described by Guo *et al*. (2012) [13]. FM^®^ 4–64 dye (N-(3-Triethylammoniumpropyl)-4-(6-(4-(Diethylamino) Phenyl) Hexatrienyl) Pyridinium Dibromide; ThermoFisher Scientific) was used as a positive membrane marker. Florescence was detected 24-hours post-transfection using a Zeiss LSM 710 AxioObserver confocal microscope with the Plan-Apochromat 40x/1.4 Oil DIC M27. Excitation wavelength for GFP and FM^®^ 4–64 was 488 and 515 nm, respectively, while emission was detected at 495–540 and 640 nm, respectively. Images were processed with Zen 2 lite blue edition (Zeiss).

### Analysis of the cell wall lignin content

Klason-lignin content of the bottom 15 cm of the dominant *Arabidopsis* inflorescence stems were determined as described by Maloney and Mansfield (2010) [14]. Briefly, stems were dried at 50°C for 48 hours and ground in a Wiley mill containing a 40-mesh screen. Thereafter, extractives were removed via acetone Soxhlet extraction at 70°C for 16 hours and ~150 mg of dry, extractive-free tissue was used to determine lignin content. Stem tissue was macerated in 72% (v/v) sulfuric acid for 2 hours, diluted with 112 ml deionized water and thereafter autoclaved at 121°C for an hour. The acid-insoluble lignin was quantified using pre-weighed medium coarseness glass crucibles, while a UV/VIS Spectrometer was used to determine the acid-soluble lignin content at 205 nm. Thioacidolysis was carried out via the method described by Robinson and Mansfield (2009), while gas chromatography was performed on a ThermoFisher TRACE 1300/SSL/FID/AS1310 GC fitted with a RTX5ms (30 m, 0.25 mm ID) capillary column [15].

### Bioinformatics analysis

*Arabidopsis* DUF1218-containing proteins were aligned with ClustalW using the BLOSUM weight matrix and the Neighbor-joining method with p-distance and pairwise deletion used to construct the phylogenetic tree with 1000 bootstrap replicates. The analysis was conducted with MEGA 5.01 [16].

Prediction of subcellular localization, signal peptide and transmembrane domains were conducted using default settings of SUBA 3 [17], Signal-3L [18] and TMHMM [19], respectively. Expression profiles for *MWL-1* and *2*, across different developmental stages, was extracted from The Bio-Analytic Resource for Plant Biology (http://bar.utoronto.ca/efp/cgi-bin/efpWeb.cgi) [20]. Co-expressed genes for *MWL-1* and *MWL-2* were queried on ATTED-II (S2 and S3 Tables, respectively), while Gene Ontology-Full was performed using BiNGO 2.44 [21] in Cytoscape 2.8.2 [22]. Finally, overrepresentation summary enrichment was performed with the REVIGO server [23].

## Results

### MWL-1and MWL-2 comparative analysis

Phylogenetic analysis of the DUF1218 family from *Arabidopsis* revealed that MWL-1 is more closely related to MWL-2 in than to the other 13 members of the family (S1A Fig). The full-length DUF1218-containing proteins varied from 163 to 257 amino acids in length. According to SUBA3 [17] which combines results from 22 prediction programs and literature, these proteins were targeted to the cell periphery (plasma membrane or extracellular). Furthermore, all proteins (except At1g52910 and At4g21310) contained a predicted signal peptide and varied in their transmembrane topology (S1A Fig). Full-length MWL-1 and MWL-2 proteins have approximately 34 and 58% amino acid identity and similarity respectively, while the domain itself (*i*.*e*. DUF1218) have approximately 49 and 71% amino acid identity and similarity, respectively. Both MWL-1 and MWL-2 are predicted to be membrane localized and share the same predicted membrane topology—the predicted mature protein containing three trans-membrane domains with the N-terminus facing the extracellular space (S1A Fig).

Expression profiles for *MWL-1* and *MWL-2* across 47 developmental stages of *Arabidopsis* growth were mined from The Bio-Analytic Resource for Plant Biology (http://bar.utoronto.ca/efp/cgi-bin/efpWeb.cgi) [20] and analyzed with Multi Experiment Viewer [24] (S1B Fig). Both proteins have ubiquitous background expression, however, there are distinct developmental phases with preferential expression for each gene. These include the stem 1^st^ node, 2^nd^ internode and flower stage 12-stamen for *MWL-1* and cauline leaf, flower stage 15, sepals and senescing leaf for *MWL-2*. In addition, notable expression of *MWL-2* occurs in the root and 2^nd^ internode of the stem, overlapping with *MWL-1* expression in the stem. *Arabidopsis* co-expression analysis on ATTED-II [10] with *MWL-1* as query revealed several cellulose, xylan and lignin-related genes (S2 Table). Gene ontology (GO) enrichment of these genes showed overrepresentation of cell wall-related terms including cell wall biogenesis, lignin catabolism, cellular polysaccharide biosynthesis, establishment of vesicle localization and xylem development (S2 Fig).

### MWL-1 and MWL-2 are targeted to the cell membrane

**Fig 1:**
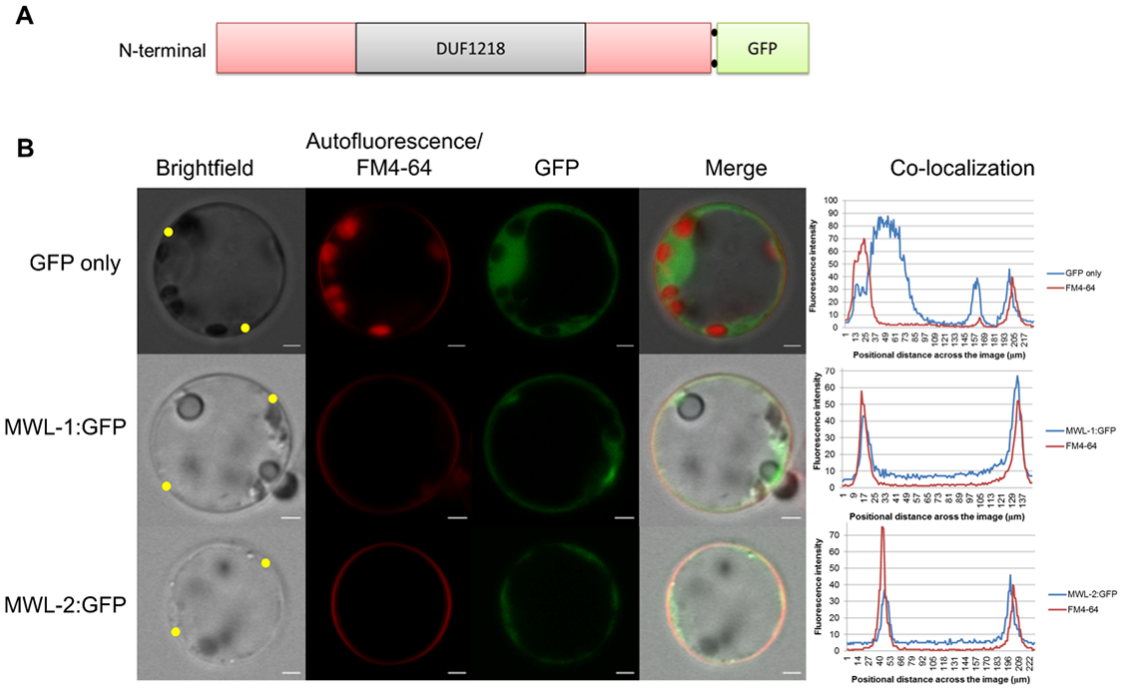
Localization of MWL-1 and MWL-2 from *Arabidopsis* at the cell periphery. A. Schematic representation of the *Arabidopsis* DUF1218-containing MWL-1 and MWL-2 proteins fused to Green Fluorescent Protein (GFP) at the C-terminal. B. Confocal microscopy of transiently expressed MWL-1 and MWL-2 with C-terminal GFP fusion in *Populus alba* × *tremula*, P717 mesophyll protoplast. The first column shows brightfield images, the second column shows autofluorescence and FM^®^ 4–64 membrane stain, the third column shows the GFP fusion signal and the fourth column shows the merge of the first three panels. Scale bar, 2 μm. The co-localization plots graphs (far right), generated with Zen 2 lite shows the co-localization intensity profile of the GFP fusion proteins and FM^®^ 4–64 across the diameter of representative protoplast cells. Yellow dots on the brightfield image are indicative of the location through which the co-localization profile was drawn.

To verify the predicted subcellular localization of MWL-1 and MWL-2, GFP was fused to the C-terminus of both MWL-1 and MWL-2. The reason for this fusion orientation was due to a predicted signal peptide at the N-terminal of both candidate proteins. The fluorescent signal in mesophyll protoplasts showed a predominant patterning at the cell periphery for both MWL-1 and MWL-2, and co-localized with the membrane marker, FM^®^ 4–64 (Fig 1). MWL-1 and MWL-2 fluorescent patterning contrasted with the GFP-only control, which was distributed throughout the cytoplasm and nucleus. The co-localization of the GFP-fusion proteins with FM^®^ 4–64 was verified using co-localization profile plot graphs across representative protoplast cells in Zen 2 lite (Zeiss) (Fig 1). In some instances, the fluorescence for MWL-1 and 2 formed foci in addition to membrane patterning after prolonged incubation, which may be indicative of vesicles or alternatively high level of expression and accumulation of the fusion proteins.

Since MWL-1 and MWL-2 are localized to the membrane, the Membrane-based Interactome Network Database (http://cas-biodb.cas.unt.edu/project/mind/search.php), based on a split-ubiquitin yeast two-hybrid screen for *Arabidopsis* membrane proteins, was interrogated for physical interactors with the candidate proteins [25]. While no interactions were detected for MWL-2, MWL-1 was found to have a strong interaction with a CYSTEINE-RICH RECEPTOR-LIKE PROTEIN KINASE19 (AT4G23270). Interestingly, CYSTEINE-RICH RECEPTOR-LIKE PROTEIN KINASE19 is predicted to contain two transmembrane domains [19] and localize to the cell membrane [17]. Overall, the results of the subcellular localization suggest that MWL-1 and MWL-2 are indeed localized to the cell membrane.

### Simultaneous Knockout of MWL-1 and MWL-2 Affects Plant Growth and Development

**Fig 2:**
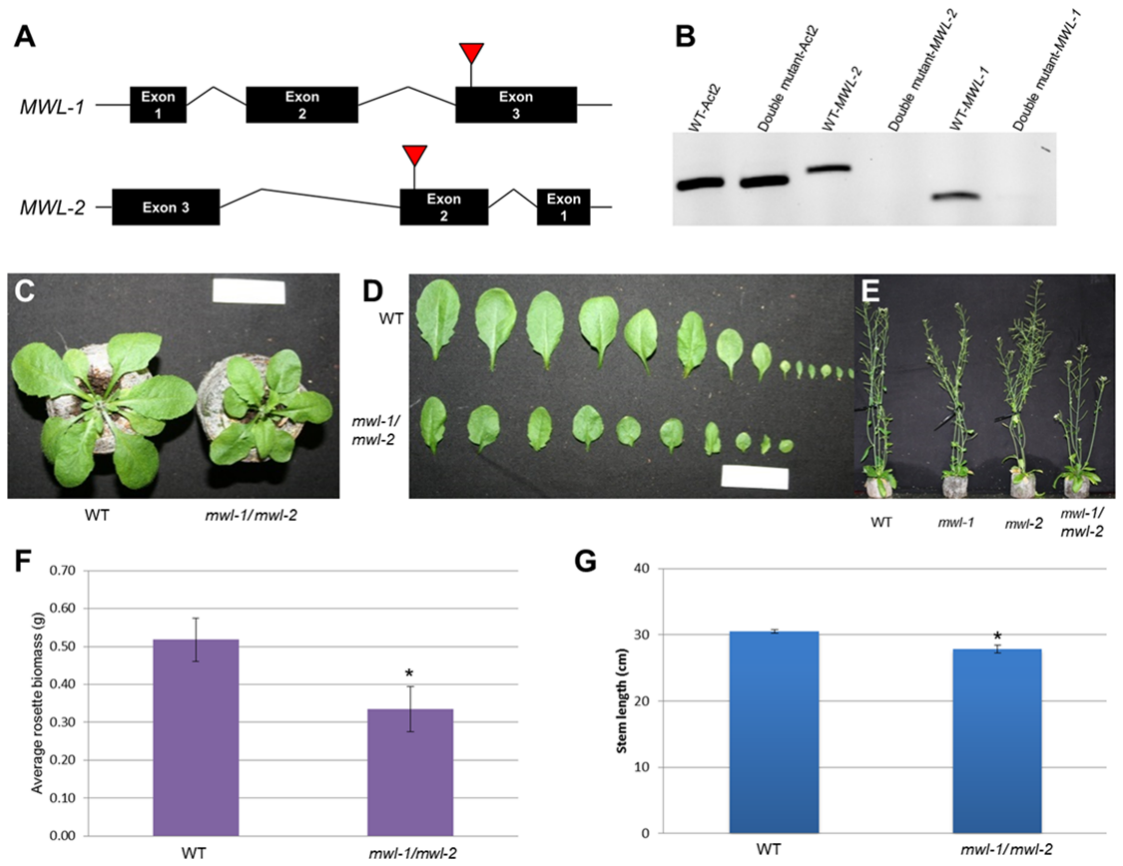
Characterization of *mwl-1* and *mwl-2* insertion alleles and overall plant growth of the *mwl-1/mwl-2* double knockout mutant. (A) Genomic structure of *MWL-1* and *MWL-2* comprises three exons and two introns showing disruption by the T-DNA in exon 3 and 2, respectively. (B) RT-PCR detected endogenous *MWL-1* and *MWL-2* transcript in the wild-type plants (WT), while endogenous transcript was absent in the homozygous double knockout mutant. Actin2 was used as an internal control gene. Position of the oligonucleotides used for the RT-PCR is indicated in A by arrows and the sequences can be found in S1 Table. (C and D). Qualitative comparison of rosette (C) and leaves (D) of double mutant relative to the WT control plants after four weeks growth. (E) Qualitative comparison of single and double knockout inflorescence stems relative to WT control. (F and G) Quantitative analysis of four-week-old rosette leaves (n = 8, F), and six-week-old inflorescence stem length (n = 65) relative to WT control (G). Scale bar, 3 cm. Error bars are standard error and significant difference from the WT based on a paired two-tailed Student’s t-test (*p*-value ≤ 0.05) is indicated by an asterisk (*).

To investigate the role of MWL-1 and MWL-2 in plant development, we identified T-DNA lines disrupting these target genes (Fig 2A). We only found a single T-DNA line for MWL-1 since an alternative line had multiple insertion gene hits. Furthermore, qRT-PCR analysis of a second line for MWL-2, showed increased expression of the endogenous gene when the insert was present in the intronic region. We therefore proceeded with the T-DNA lines with the insertions in exon 3 and 2 for MWL-1 and 2, respectively (Fig 2A). RT-PCR analysis of the homozygous T-DNA lines failed to detect endogenous (uninterrupted) gene transcript in these lines (S3A and S4A Figs). These plants showed no discernable differences in terms of rosette size and stem length, and were phenotypically indistinguishable from the WT control (S3C, S3D, S4C and S4D Figs). Furthermore, overexpression of either gene under the control of the constitutive 35 CaMV promoter (confirmed by semi-quantitative PCR, S3B and S4B Figs) also revealed no discernable phenotypic differences between the wild-type control and transgenic plants (S3C, S3F, S4C and S4F Figs). Since these two genes are closely related and could act redundantly in the single-mutant lines, double mutant plants were generated by crossing the T-DNA lines (*mwl-1 x mwl-2)*. Positive double mutant lines (*mwl-1/mwl-2*) were selected on appropriate selection media and homozygosity of the lines was initially confirmed by genomic PCR (see Materials and Methods) and later confirmed by RT-PCR, which failed to detect *MWL-1* and *MWL-2* endogenous gene transcripts in the homozygous double mutant plants (Fig 2B). *mwl-1/mwl-2* showed a reduction in the overall rosette size (leaf size and number) and a significant decrease in fresh weight of the rosette leaves at four weeks relative to the control (paired two-tailed Student’s t-test, *p*-value ≤ 0.05, n = 8) (Fig 2C, 2D and 2F). The *mwl-1/mwl-2* plants also showed a significant reduction in plant height (*ca*. 9%) in comparison to the WT control after six weeks (paired two-tailed Student’s t-test, *p*-value ≤ 0.05, n = 65) (Fig 2E and 2G). No obvious secondary cell wall defects (*i*.*e*. irregular xylem or staining intensity) were observed in the overexpression, single and double knockout stem cross-sections stained with phloroglucinol (S5 Fig).

### The double knockout, *mwl-1/mwl-2*, affects lignin content and S/G monomer ratio

**Fig 3:**
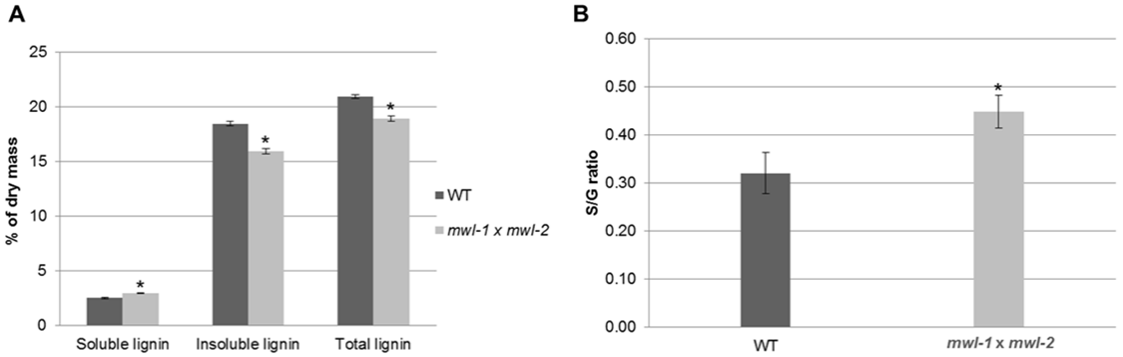
Klason lignin content and syringyl/guaiacyl (S/G) monomer ratio of *mwl-1/mwl-2* relative to the wild-type (WT) control plants. (A) Soluble, insoluble and total lignin content as percentage of extracted dry mass from WT and double mutant stems. (B) S/G monomer ratio of WT and double mutant plants. Analysis conducted on the bottom 15 cm of the dominant inflorescent stems of plants grown in soil for six weeks. Data represents three biological replicates each containing bulked stem tissues of approximately 24 plants. Error bars indicate the standard error and significant differences from the WT is based on paired two-tailed Student’s t-test (*p*-value ≤ 0.05) is indicated by asterisk (*).

To investigate the effect the genes may have on cell wall traits, lignin (soluble and insoluble) content was analyzed from the bottom half of extractive-free dried stems of the knockout and overexpression lines following six weeks growth in soil. There was no discernible change/trend seen in the lignin content of the overexpression lines and the single T-DNA lines analyzed relative to the WT control (S4 Table). However, the double knockout mutant showed a significant decrease in insoluble and total lignin (*ca*.16 and 11%, respectively) and significant increase in the soluble lignin (*ca*. 18%) relative to the WT control plants that were grown in parallel under similar conditions (Fig 3A and S4 Table). Next, the effect of the double knockout on lignin composition was investigated by analysis of the lignin monomer content by thioacidolysis, which revealed a significant increase in the S/G ratio in the double mutant line relative to the wildtype control (ca. 40% fold change, 0.32 vs. 0.45, Fig 3B). Changes in the cell wall chemistry through simultaneous perturbation of both *MWL-1* and *MWL-2* were in agreement with GO enrichment for *MWL-1* co-expressed genes (S2 Table and S2 Fig). The inability to detect the changes in the lignin content observed in the double mutant through the histochemical staining of stem cross-sections with phloroglucinol may be due to the moderate decrease in the total lignin (Fig 3), or a change in the soluble cell wall phenolics which were not quantified (phloroglucinol stains aldehydes, and therefore if compensatory changes occur in these plants in response to the double knockout, it would not be detected by the histochemical stain).

## Discussion

The *Arabidopsis thaliana* genome encodes 15 members belonging to the DUF1218 protein family (S1A Fig) and the importance of several members in plant development and physiology have already been illustrated in the literature [3–6,9]. The aim of the current study was to functionally characterize another member of the DUF1218 family, *MWL-1*, as well as the most closely related member *At4g19370* (*MWL-2*) and investigate the possible role of these proteins in cell wall biology.

Expression profiles (S1B Fig) and co-expressed genes (S2 and S3 Tables) are distinct for the two genes, which may suggest sub/neofunctionalization. Despite this, basal expression within a common tissue (e.g. inflorescent stem tissue) may contribute to functional redundancy, especially since these proteins have the DUF1218 in common, identical subcellular localization and predicted transmembrane topology (Fig 1 and S1A Fig). Indeed, observed phenotypic changes in the double mutant (Figs 2C–2G and 3) and not in the single mutants suggest functional redundancy for *MWL-1* and *MWL-2* genes. The absence of discernible phenotype in the overexpression lines suggest that wild type expression of either *MWL-1* or *MWL-2* is sufficient for effective functioning, or that the effect of overexpression is limited by the abundance of interacting partners.

Decreases in lignin content have been shown to impede plant growth and development [26,27]. Therefore, in the case of the double knockout, a moderate decrease in lignin content was also associated with moderate decrease in plant growth. Moreover, smaller leaf area and fewer leaves may explain the reduction in plant height due to accompanied reduction of total photosynthetic capacity, which may have implications for cell wall biopolymer synthesis due to carbon availability.

Based on the evidence, it is attractive to hypothesize that MWL-1 plays a role in signalling or transport during lignin biosynthesis. This may be further supported by the physical interaction of MWL-1 with CYSTEINE-RICH RECEPTOR-LIKE PROTEIN KINASE19 (CRK19) which was detected using yeast two-hybrid screen for *Arabidopsis* membrane proteins [25]. CRK19, in turn, is co-expressed with several signalling and transporter proteins e.g. *ARABIDOPSIS THALIANA* ATP-BINDING CASSETTE G36 (-2), and Plant PDR ABC-type transporter family protein (At1g59870). Recently, it was shown that *At*ABC-G29 functions as a putative monolignol transporter for *p*-coumaryl alcohol, which gives rise to p-hydroxyphenyl (H) units [28]. However, while six of the twelve *Arabidopsis* ABC transporters are located at the plasma membrane [29,30], the identity of other monolignol transporters remains elusive.

Recently, it was shown that a T-DNA knockout line of At4g27435 resulted in an increase in lignin content in the inflorescent stem tissue (Mewalal *et al*., unpublished). MWL-1/2 and At4g27435 have predicted opposite orientation at the membrane *i*.*e*. N-terminal orientated on the outside and inside of the cell respectively for the mature proteins. Given the opposite effects of these oppositely oriented proteins, these proteins may act antagonistically to each other. However, further experimentation is necessary to validate such a hypothesis.

The current study motivates further investigation into the cell wall functioning of DUF1218 members *e*.*g*. the impact in a woody system such as *Populus* would be interesting. Nevertheless, we describe the functional redundancy of the DUF1218-containing MWL-1 and MWL-2 proteins and confirm their roles in secondary cell wall chemistry.

## Supporting Information

S1 Fig Phylogenetic and bioinformatics analysis of all members of the *Arabidopsis* domain of unknown function 1218 (DUF1218) family, and expression profiling of the candidate members, MODIFYING WALL LIGNIN-1 (MWL-1, At1g31720) and MWL-2 (At4g19370). (A) Neighbor-joining phylogenetic tree of *Arabdiopsis* DUF1218-containing proteins. ClustalW was used to align protein sequences from TAIR and the alignment thereafter used to construct the tree using p-distance and pairwise deletion with 1000 bootstrap replicates in MEGA5 [16]. Prediction of subcellular localization, signal peptide and number of transmembrane domains was done using SUBA3 [31], Signal-3L [18] and TMHMM [19] respectively, with default settings. Highlighted in pink are the related MWL-1 and 2 sequences. (B) *Arabidopsis* expression profiles for *MWL-1* and *MWL-2* across different tissues during development, exctracted from The Bio-Analytic Resource for Plant Biology (http://bar.utoronto.ca/welcome.htm) [20]. Preferential expression is seen at distinct developmental stages, however, there is overlap in the secondary cell wall depositing, 2^nd^ internode region.
(DOCX)

S2 Fig Gene ontology enrichment of MWL-1 top 300 co-expressed genes in *Arabidopsis*. Co-expressed genes were extracted from ATTED-II [10]. GO-full was conducted in Cytoscape 2.8.2 [22] using BiNGO 2.44 [21], while overrepresentation summary enrichment was performed with the REVIGO server [23].
(DOCX)

S3 Fig Phenotypic analysis of At1g31720 (*MWL-1*) single T-DNA knockout line mutants and *MWL-1* overexpression lines. (A) RT-PCR detection of endogenous *MWL-1* transcript in the wildtype (WT) plants and absence in the single knockout mutant. (B) Semi-quantitative RT-PCR analysis of *MWL-1* overexpression lines 1 to 3 showing detection of MWL-1 transgene in the transgenic lines. Actin2 was used as a control gene and RT-PCR was performed on cDNA from stem tissue. Actin2 and *MWL-1* gene-specific oligonucleotide sequences can be found in S1 Table. Rosette size (C) and mass (D) of *MWL-1* single T-DNA knockout line and overexpression lines 1–3 relative to (WT) control line at four weeks. Qualitative (E) and quantitative (F) stem length of *MWL-1* single T-DNA knockout line and overexpression lines relative to WT control at six weeks. For rosette mass n = 3 and for quantitative stem length n = 66. Error bars indicate the standard error. Scale bar, 3 cm. Based on a two-tailed Student’s t-test (*P*-value ≤ 0.05) no significant differences were seen in the growth and development of the single mutant and transgenic OE lines in comparison to the WT controls.
(DOCX)

S4 Fig Phenotypic analysis of At4g19370 (MWL-2) single T-DNA knockout line mutants and MWL-2 overexpression lines. (A) RT-PCR detection of endogenous *MWL-2* transcript in the wildtype (WT) plants and absence in the single knockout mutant. (B) Semi-quantitative RT-PCR analysis of *MWL-2* overexpression lines 1 to 3 showing detection of *MWL-2* transgene in the transgenic lines except for OE1 which could be indicative of positional effect (position in the genome), or co-suppression dominant repression. Actin2 was used as a control gene and RT-PCR was performed on stem tissue. Actin2 and MWL-2 gene-specific oligonucleotide sequences can be found in S1 Table. Rosette size (C) and mass (D) of *MWL-2* single T-DNA knockout line and overexpression lines 1–3 relative to (WT) control line at four weeks. Qualitative (E) and quantitative (F) stem length of *MWL-2* single T-DNA knockout line and overexpression lines relative to WT control at six weeks. For rosette mass n = 3 and for quantitative stem length n = 66. Error bars indicate the standard error while significant difference from the WT based on a two-tailed Student’s t-test (*P*-value ≤ 0.05) is indicated by *. Scale bar, 3 cm. No significant differences were seen in the growth and development of the single mutant and transgenic OE lines in comparison to the WT controls except for OE-Line 2.
(DOCX)

S5 Fig Transverse sections of six-week-old stem tissue stained with phloroglucinol from At1g31720 (MWL-1) and At4g19370 (MWL-2) T-DNA knockout mutant and overexpression (OE) lines. Transverse sections from wildtype (WT) (A), *At1g31720* mutant (B), *At4g19370* mutant (C), *At1g31720* x *At4g19370* double knockdown mutant (D), OEAt1g31720 line 1 (E), line 2 (F), line 3 (G), OEAt4g19370 line 1(H), line 2 (I), line 3 (J). Scale bar, 100μm (indicated in red). No discernible differences were seen in the transverse sections of the single and double mutant as well as the transgenic OE lines in comparison to the WT controls.
(DOCX)

S1 Table List of oligonucleotides used in the study. (DOCX)

S2 Table Top 300 *Arabidopsis* co-expressed genes for *MWL-1* (*At1g31720*) from ATTED-II represented as MR value. (DOCX)

S3 Table Top 300 *Arabidopsis* co-expressed genes for *MWL-2* (*At4g19370*) from ATTED-II represented as MR value. (DOCX)

S4 Table Structural cell wall carbohydrates and lignin content from MWL-1 and MWL-2 overexpression, single and double knockout lines compared to its respective wildtype (WT) control. (DOCX)

## References

[1] Wolf S, Hématy K, Höfte H. Growth control and cell wall signaling in plants. Annu Rev Plant Biol. 2012;63: 381–407. 10.1146/annurev-arplant-042811-105449 22224451

[2] Kalluri UC, Yin H, Yang X, Davison BH. Systems and synthetic biology approaches to alter plant cell walls and reduce biomass recalcitrance. Plant Biotechnol J. 2014;12(9): 1207–16. 10.1111/pbi.12283 25363806 PMC4265275

[3] Ubeda-Tomas S, Edvardsson E, Eland C, Singh SK, Zadik D, Aspeborg H, et al Genomic-assisted identification of genes involved in secondary growth in *Arabidopsis* utilising transcript profiling of poplar wood‐forming tissues. Physiol Plant. 2006;129(2): 415–28.

[4] Persson S, Wei H, Milne J, Page GP, Somerville CR. Identification of genes required for cellulose synthesis by regression analysis of public microarray data sets. Proc Natl Acad Sci U S A. 2005;102(24): 8633 15932943 10.1073/pnas.0503392102 PMC1142401

[5] Roschzttardtz H, Paez-Valencia J, Dittakavi T, Jali S, Reyes F, Baisa G, et al The VASCULATURE COMPLEXITY AND CONNECTIVITY (VCC) gene encodes a plant-specific protein required for embryo provasculature development. Plant Physiol. 2014:pp. 114.246314 10.1104/pp.114.246314 PMC4213116 25149602

[6] Huang X, Wang G, Shen Y, Huang Z. The wheat gene TaST can increase the salt tolerance of transgenic Arabidopsis. Plant Cell Rep. 2012;31(2): 1–9. 21993814 10.1007/s00299-011-1169-9

[7] Mandadi KK, Scholthof KBG. Characterization of a viral synergism in the monocot *Brachypodium* reveals distinctly altered host molecular processes associated with disease. Plant Physiol. 2012;160(3):1432–52. 10.1104/pp.112.204362 22961132 PMC3490591

[8] Oda S, Kaneko F, Yano K, Fujioka T, Masuko H, Park JI, et al Morphological and gene expression analysis under cool temperature conditions in rice anther development. Genes Genet Syst. 2010;85(2): 107–20. 20558897 10.1266/ggs.85.107

[9] Brown DM, Zeef LAH, Ellis J, Goodacre R, Turner SR. Identification of novel genes in *Arabidopsis* involved in secondary cell wall formation using expression profiling and reverse genetics. The Plant Cell Online. 2005;17(8): 2281. 10.1105/tpc.105.031542 PMC1182489 15980264

[10] Obayashi T, Hayashi S, Saeki M, Ohta H, Kinoshita K. ATTED-II provides coexpressed gene networks for *Arabidopsis*. Nucleic Acids Res. 2009;37(suppl 1): D987–D91. 18953027 10.1093/nar/gkn807 PMC2686564

[11] Curtis MD, Grossniklaus U. A gateway cloning vector set for high-throughput functional analysis of genes in planta. Plant Physiol. 2003;133(2): 462–9. 14555774 10.1104/pp.103.027979 PMC523872

[12] Clough SJ, Bent AF. Floral dip: a simplified method for Agrobacterium-mediated transformation of *Arabidopsis thaliana*. The Plant Journal. 1998;16(6): 735–43. 10069079 10.1046/j.1365-313x.1998.00343.x

[13] Guo J, Morrell-Falvey JL, Labbé JL, Muchero W, Kalluri UC, Tuskan GA, et al Highly efficient isolation of Populus mesophyll protoplasts and its application in transient expression assays. PLoS One. 2012 9 13 10.1371/journal.pone.0044908 PMC3441479 23028673

[14] Maloney VJ, Mansfield SD. Characterization and varied expression of a membrane-bound endo-β-1, 4-glucanase in hybrid poplar. Plant Biotechnol J. 2010;8(3): 294–307. 10.1111/j.1467-7652.2009.00483.x 20070872

[15] Robinson AR, Mansfield SD. Rapid analysis of poplar lignin monomer composition by a streamlined thioacidolysis procedure and near-infrared reflectance-based prediction modeling. The Plant Journal. 2009;58(4): 706–14. 10.1111/j.1365-313X.2009.03808.x 19175772

[16] Tamura K, Peterson D, Peterson N, Stecher G, Nei M, Kumar S. MEGA5: molecular evolutionary genetics analysis using maximum likelihood, evolutionary distance, and maximum parsimony methods. Mol Biol Evol. 2011;28(10): 2731–9. 10.1093/molbev/msr121 21546353 PMC3203626

[17] Tanz SK, Castleden I, Hooper CM, Vacher M, Small I, Millar HA. SUBA3: a database for integrating experimentation and prediction to define the SUBcellular location of proteins in *Arabidopsis*. Nucleic Acids Res. 2012: gks1151. 10.1093/nar/gks1151 PMC3531127 23180787

[18] Shen H-B, Chou K-C. Signal-3L: A 3-layer approach for predicting signal peptides. Biochem Biophys Res Commun. 2007;363(2): 297–303. 17880924 10.1016/j.bbrc.2007.08.140

[19] Krogh A, Larsson B, Von Heijne G, Sonnhammer EL. Predicting transmembrane protein topology with a hidden Markov model: application to complete genomes. J Mol Biol. 2001;305(3): 567–80. 11152613 10.1006/jmbi.2000.4315

[20] Schmid M, Davison TS, Henz SR, Pape UJ, Demar M, Vingron M, et al A gene expression map of *Arabidopsis thaliana* development. Nat Genet. 2005;37(5): 501–6. 15806101 10.1038/ng1543

[21] Maere S, Heymans K, Kuiper M. BiNGO: a Cytoscape plugin to assess overrepresentation of gene ontology categories in biological networks. Bioinformatics. 2005;21(16): 3448–9. 15972284 10.1093/bioinformatics/bti551

[22] Smoot ME, Ono K, Ruscheinski J, Wang P-L, Ideker T. Cytoscape 2.8: new features for data integration and network visualization. Bioinformatics. 2011;27(3): 431–2. 10.1093/bioinformatics/btq675 21149340 PMC3031041

[23] Supek F, Bošnjak M, Škunca N, Šmuc T. REVIGO summarizes and visualizes long lists of gene ontology terms. PloS one. 2011;6(7): e21800 10.1371/journal.pone.0021800 21789182 PMC3138752

[24] Saeed A, Sharov V, White J, Li J, Liang W, Bhagabati N, et al TM4: a free, open-source system for microarray data management and analysis. BioTechniques. 2003;34(2): 374 12613259 10.2144/03342mt01

[25] Jones AM, Xuan Y, Xu M, Wang R-S, Ho C-H, Lalonde S, et al Border Control—A Membrane-Linked Interactome of *Arabidopsis*. Science. 2014;344(6185): 711–6. 10.1126/science.1251358 24833385

[26] Derikvand MM, Sierra JB, Ruel K, Pollet B, Do C-T, Thévenin J, et al Redirection of the phenylpropanoid pathway to feruloyl malate in *Arabidopsis* mutants deficient for cinnamoyl-CoA reductase 1. Planta. 2008;227(5): 943–56. 18046574 10.1007/s00425-007-0669-x

[27] Do C-T, Pollet B, Thévenin J, Sibout R, Denoue D, Barrière Y, et al Both caffeoyl Coenzyme A 3-O-methyltransferase 1 and caffeic acid O-methyltransferase 1 are involved in redundant functions for lignin, flavonoids and sinapoyl malate biosynthesis in *Arabidopsis*. Planta. 2007;226(5): 1117–29. 17594112 10.1007/s00425-007-0558-3

[28] Alejandro S, Lee Y, Tohge T, Sudre D, Osorio S, Park J, et al AtABCG29 is a monolignol transporter involved in lignin biosynthesis. Curr Biol. 2012;22(13): 1207–12. 10.1016/j.cub.2012.04.064 22704988

[29] Sibout R, Höfte H. Plant cell biology: the ABC of monolignol transport. Curr Biol. 2012;22(13): R533–R5. 10.1016/j.cub.2012.05.005 22790004

[30] Dunkley TP, Hester S, Shadforth IP, Runions J, Weimar T, Hanton SL, et al Mapping the *Arabidopsis* organelle proteome. Proc Natl Acad Sci U S A. 2006;103(17): 6518–23. 16618929 10.1073/pnas.0506958103 PMC1458916

[31] Hooper CM, Tanz SK, Castleden IR, Vacher MA, Small ID, Millar AH. SUBAcon: a consensus algorithm for unifying the subcellular localization data of the *Arabidopsis* proteome. Bioinformatics. 2014;30(23): 3356–64. 10.1093/bioinformatics/btu550 25150248

