# Supplementary material for: The Arabidopsis Domain of Unknown Function 1218 (DUF1218) Containing Proteins, MODIFYING WALL LIGNIN-1 and 2 (At1g31720/MWL-1 and At4g19370/MWL-2) Function Redundantly to Alter Secondary Cell Wall Lignin Content
Source: PLoS One. 2016 Mar 1;11(3):e0150254. doi: 10.1371/journal.pone.0150254 (PMC4773003; doi:10.1371/journal.pone.0150254)
Supplement: S1 Fig — (A) Neighbor-joining phylogenetic tree of Arabdiopsis DUF1218-containing proteins. ClustalW was used to align protein sequences from TAIR and the alignment thereafter used to construct the tree using p-distance and pairwise deletion with 1000 bootstrap replicates in MEGA5 [16]. Prediction of subcellular localization, signal peptide and number of transmembrane domains was done using SUBA3 [31], Signal-3L [18] and TMHMM [19] respectively, with default settings. Highlighted in pink are the related MWL-1 and 2 sequences. (B) Arabidopsis expression profiles for MWL-1 and MWL-2 across different tissues during development, exctracted from The Bio-Analytic Resource for Plant Biology (http://bar.utoronto.ca/welcome.htm) [20]. Preferential expression is seen at distinct developmental stages, however, there is overlap in the secondary cell wall depositing, 2nd internode region. (DOCX) [file pone.0150254.s001.docx]

 
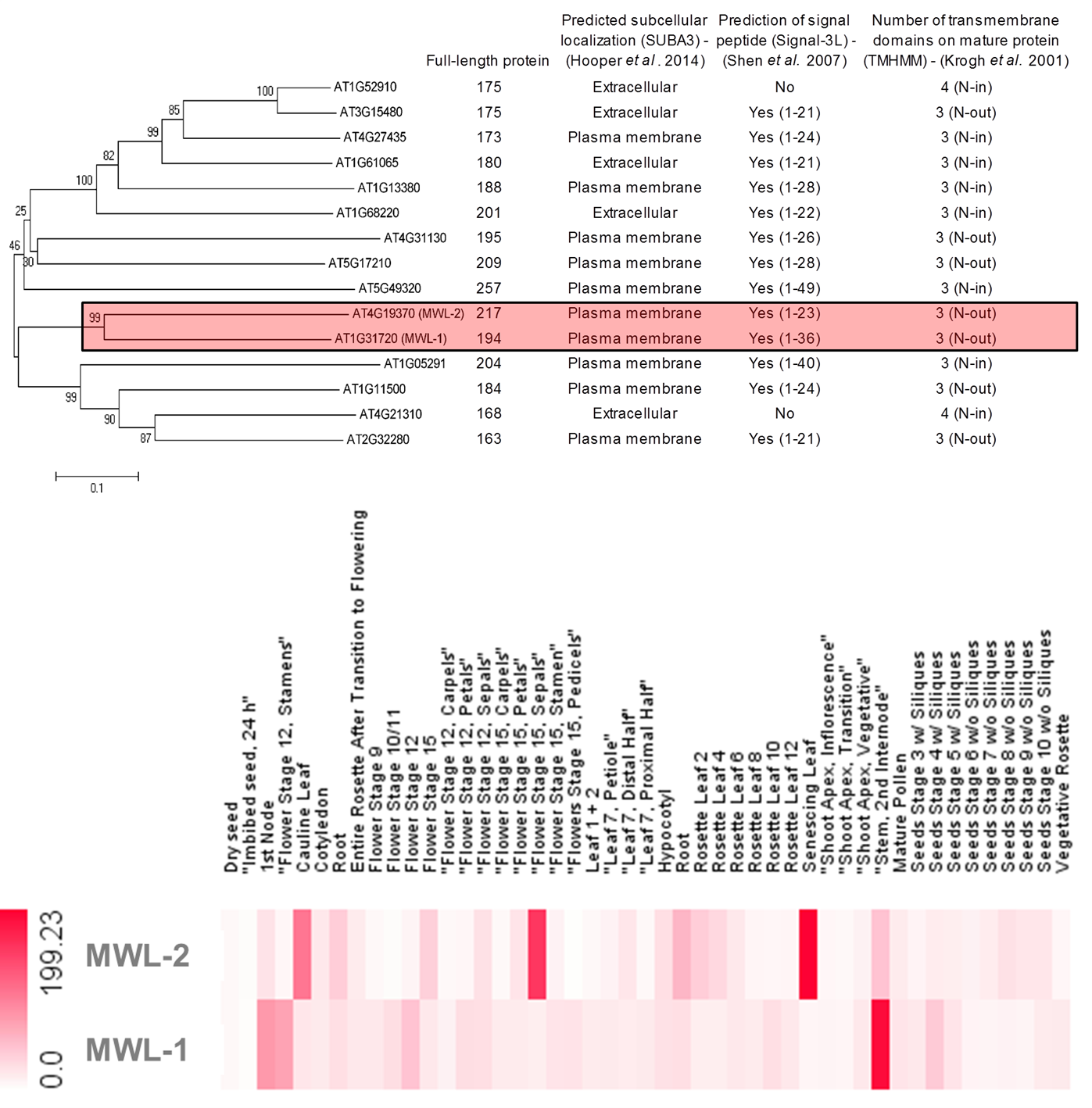


**B**

**A**

S1 Fig. Comparative analysis of all members of the *Arabidopsis* domain of unknown function 1218 (DUF1218) family and expression profiling of the candidate members, MODIFYING WALL LIGNIN-1 (*MWL-1*, At1g31720) and *MWL-2* (At4g19370).

A. Neighbor-joining phylogenetic tree of *Arabdiopsis* DUF1218-containing proteins. ClustalW was used to align protein sequences from TAIR and the alignment thereafter used to construct the tree using p-distance and pairwise deletion with 1000 bootstrap replicates in MEGA5 [[1](#_ENREF_1)]. Prediction of subcellular localization, signal peptide and number of transmembrane domains was done using SUBA3 [[2](#_ENREF_2)], Signal-3L [[3](#_ENREF_3)] and TMHMM [[4](#_ENREF_4)] respectively with default settings. Highlighted in pink are the related MWL-1 and 2 sequences. B. *Arabidopsis* expression profiles for *MWL-1* and *MWL-2* across different tissues during development, exctracted from The Bio-Analytic Resource for Plant Biology (<http://bar.utoronto.ca/welcome.htm>) [[5](#_ENREF_5)]. Preferential expression is seen at distinct developmental stages, however there is overlap in the secondary cell wall depositing, 2^nd^ internode region.

# References

1. Tamura K, Peterson D, Peterson N, Stecher G, Nei M, et al. MEGA5: Molecular evolutionary genetics analysis using maximum likelihood, evolutionary distance, and maximum parsimony methods. Mol Biol and Evol. 2011. 28: 2731-2739.

2. Hooper CM, Tanz SK, Castleden IR, Vacher MA, Small ID, et al. (2014) SUBAcon: a consensus algorithm for unifying the subcellular localization data of the *Arabidopsis* proteome. Bioinformatics. 2014. 30: 3356-3364.

3. Shen H-B, Chou K-C Signal-3L: A 3-layer approach for predicting signal peptides. Biochemical and Biophysical Research Comm. 2007. 363: 297-303.

4. Krogh A, Larsson B, Von Heijne G, Sonnhammer EL (2001) Predicting transmembrane protein topology with a hidden Markov model: application to complete genomes. J. Mol. Biol. 2001. 305: 567-580.

5. Schmid M, Davison TS, Henz SR, Pape UJ, Demar M, et al. (2005) A gene expression map of *Arabidopsis thaliana* development. Nature Genetics. 2005. 37: 501-506.
