# Supplementary material for: The Arabidopsis Domain of Unknown Function 1218 (DUF1218) Containing Proteins, MODIFYING WALL LIGNIN-1 and 2 (At1g31720/MWL-1 and At4g19370/MWL-2) Function Redundantly to Alter Secondary Cell Wall Lignin Content
Source: PLoS One. 2016 Mar 1;11(3):e0150254. doi: 10.1371/journal.pone.0150254 (PMC4773003; doi:10.1371/journal.pone.0150254)
Supplement: S2 Fig — Co-expressed genes were extracted from ATTED-II [10]. GO-full was conducted in Cytoscape 2.8.2 [22] using BiNGO 2.44 [21], while overrepresentation summary enrichment was performed with the REVIGO server [23]. (DOCX) [file pone.0150254.s002.docx]

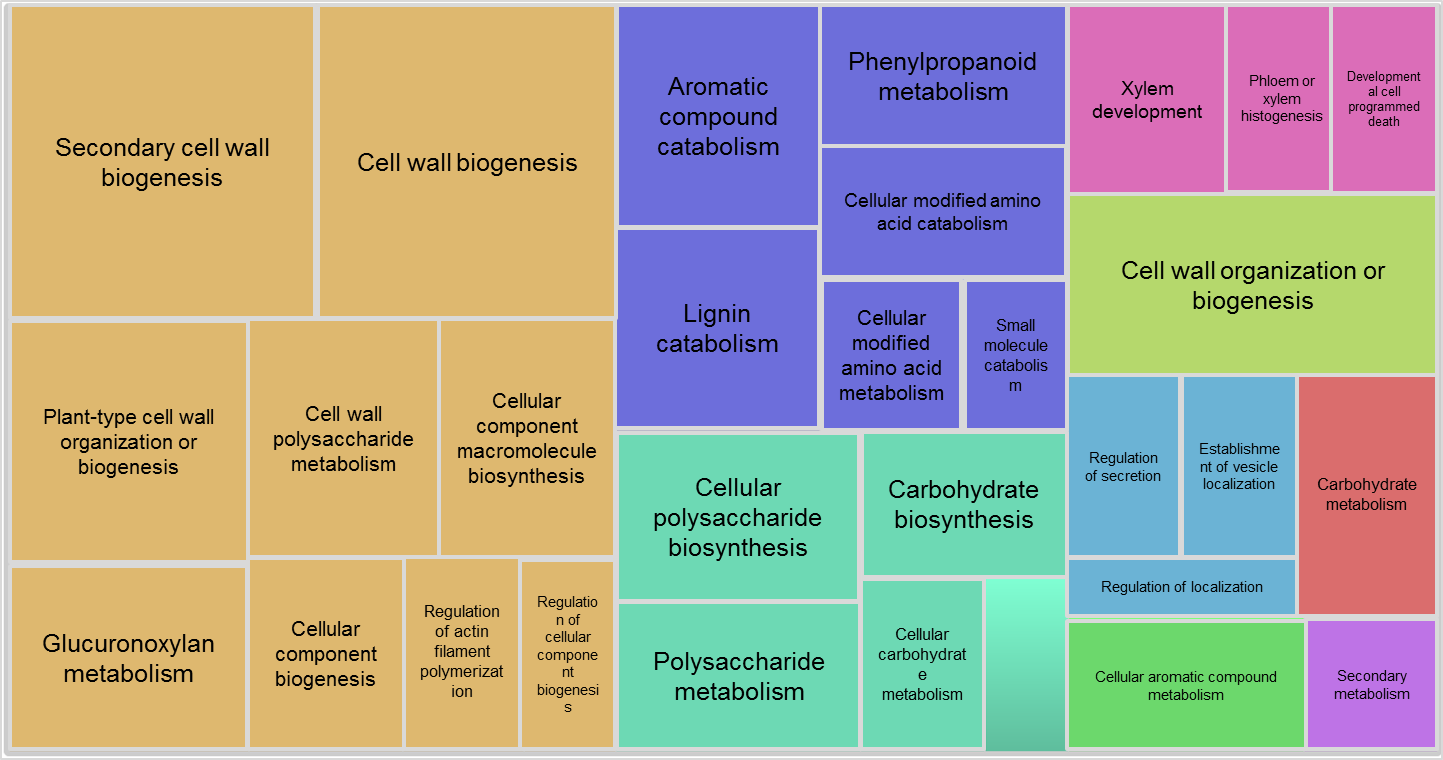


S2 Fig. Gene ontology enrichment of MWL-1 top 300 co-expressed genes in *Arabidopsis*.

Co-expressed genes were extracted from ATTED-II [1]. GO-full was conducted in Cytoscape 2.8.2 [2] using BiNGO 2.44 [3], while overrepresentation summary enrichment was performed with the REVIGO server [4].

# References

1. Obayashi T, Hayashi S, Saeki M, Ohta H, Kinoshita K. ATTED-II provides coexpressed gene networks for *Arabidopsis*. Nucleic Acids Res. 2009. 37: D987-D991.

2. Smoot ME, Ono K, Ruscheinski J, Wang P-L, Ideker T. Cytoscape 2.8: new features for data integration and network visualization. Bioinformatics. 2011. 27: 431-432.

3. Maere S, Heymans K, Kuiper M. BiNGO: a Cytoscape plugin to assess overrepresentation of gene ontology categories in biological networks. Bioinformatics. 2005. 21: 3448-3449.

4. Supek F, Bošnjak M, Škunca N, Šmuc T. REVIGO summarizes and visualizes long lists of gene ontology terms. PloS One. 2011. 6: e21800.
