# Supplementary material for: The Arabidopsis Domain of Unknown Function 1218 (DUF1218) Containing Proteins, MODIFYING WALL LIGNIN-1 and 2 (At1g31720/MWL-1 and At4g19370/MWL-2) Function Redundantly to Alter Secondary Cell Wall Lignin Content
Source: PLoS One. 2016 Mar 1;11(3):e0150254. doi: 10.1371/journal.pone.0150254 (PMC4773003; doi:10.1371/journal.pone.0150254)
Supplement: S3 Fig — (A) RT-PCR detection of endogenous MWL-1 transcript in the wildtype (WT) plants and absence in the single knockout mutant. (B) Semi-quantitative RT-PCR analysis of MWL-1 overexpression lines 1 to 3 showing detection of MWL-1 transgene in the transgenic lines. Actin2 was used as a control gene and RT-PCR was performed on cDNA from stem tissue. Actin2 and MWL-1 gene-specific oligonucleotide sequences can be found in S1 Table. Rosette size (C) and mass (D) of MWL-1 single T-DNA knockout line and overexpression lines 1–3 relative to (WT) control line at four weeks. Qualitative (E) and quantitative (F) stem length of MWL-1 single T-DNA knockout line and overexpression lines relative to WT control at six weeks. For rosette mass n = 3 and for quantitative stem length n = 66. Error bars indicate the standard error. Scale bar, 3 cm. Based on a two-tailed Student’s t-test (P-value ≤ 0.05) no significant differences were seen in the growth and development of the single mutant and transgenic OE lines in comparison to the WT controls. (DOCX) [file pone.0150254.s003.docx]

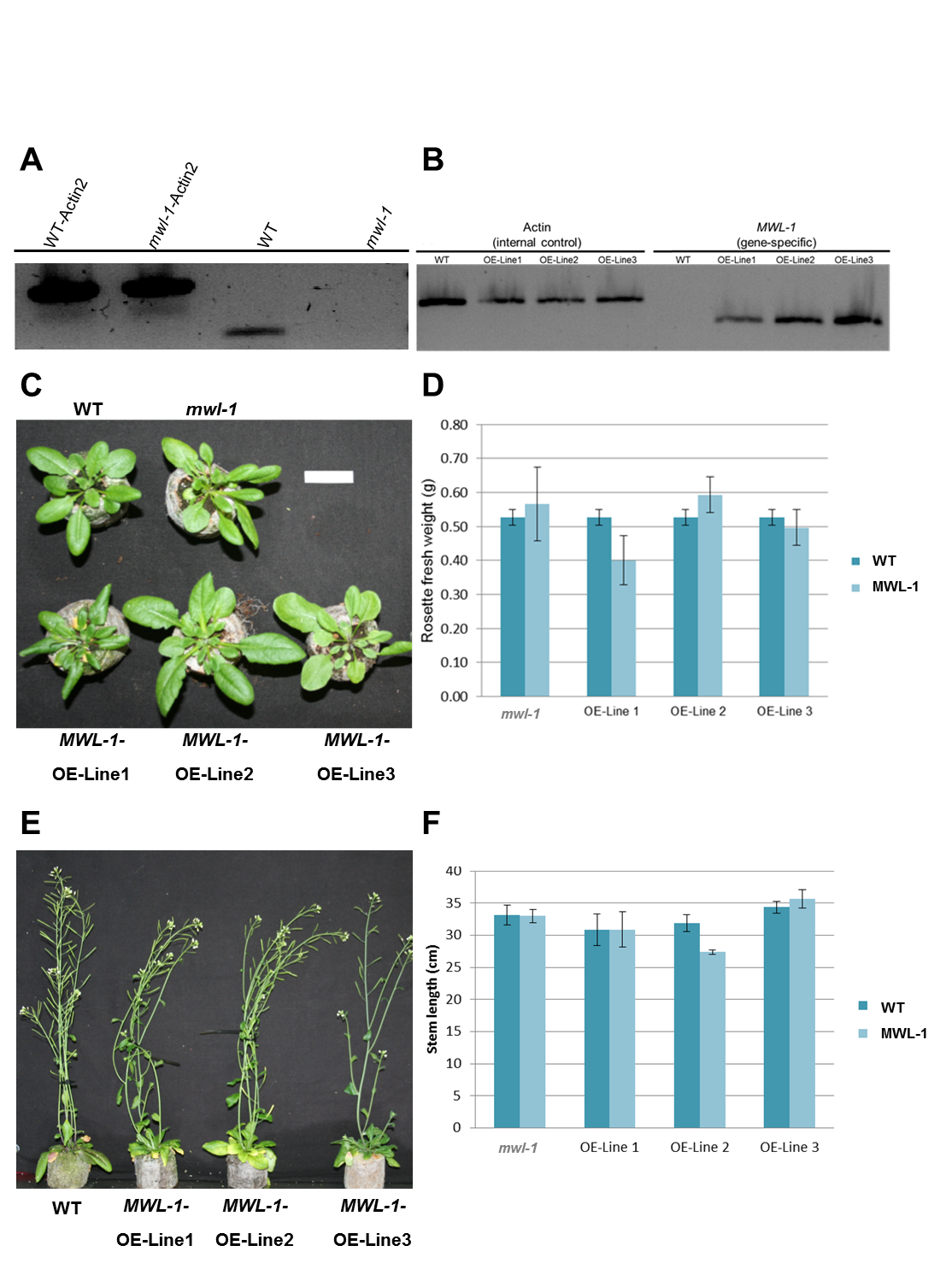


S3 Fig. Phenotypic analysis of At1g31720 (*MWL-1*) single T-DNA knockout line mutants and *MWL-1* overexpression lines.

(A) RT-PCR detection of endogenous *MWL-1* transcript in the wildtype (WT) plants and absence in the single knockout mutant. (B) Semi-quantitative RT-PCR analysis of *MWL-1* overexpression lines 1 to 3 showing detection of MWL-1 transgene in the transgenic lines. Actin2 was used as a control gene and RT-PCR was performed on cDNA from stem tissue. Actin2 and *MWL-1* gene-specific oligonucleotide sequences can be found in Table S1. Rosette size (C) and mass (D) of *MWL-1* single T-DNA knockout line and overexpression lines 1-3 relative to (WT) control line at four weeks. Qualitative (E) and quantitative (F) stem length of *MWL-1* single T-DNA knockout line and overexpression lines relative to WT control at six weeks. For rosette mass n = 3 and for quantitative stem length n = 66. Error bars indicate the standard error. Scale bar, 3 cm. Based on a two-tailed Student’s t-test (*P*-value ≤ 0.05) no significant differences were seen in the growth and development of the single mutant and transgenic OE lines in comparison to the WT controls.
