# Supplementary material for: The Arabidopsis Domain of Unknown Function 1218 (DUF1218) Containing Proteins, MODIFYING WALL LIGNIN-1 and 2 (At1g31720/MWL-1 and At4g19370/MWL-2) Function Redundantly to Alter Secondary Cell Wall Lignin Content
Source: PLoS One. 2016 Mar 1;11(3):e0150254. doi: 10.1371/journal.pone.0150254 (PMC4773003; doi:10.1371/journal.pone.0150254)
Supplement: S5 Fig — Transverse sections from wildtype (WT) (A), At1g31720 mutant (B), At4g19370 mutant (C), At1g31720 x At4g19370 double knockdown mutant (D), OEAt1g31720 line 1 (E), line 2 (F), line 3 (G), OEAt4g19370 line 1(H), line 2 (I), line 3 (J). Scale bar, 100μm (indicated in red). No discernible differences were seen in the transverse sections of the single and double mutant as well as the transgenic OE lines in comparison to the WT controls. (DOCX) [file pone.0150254.s005.docx]

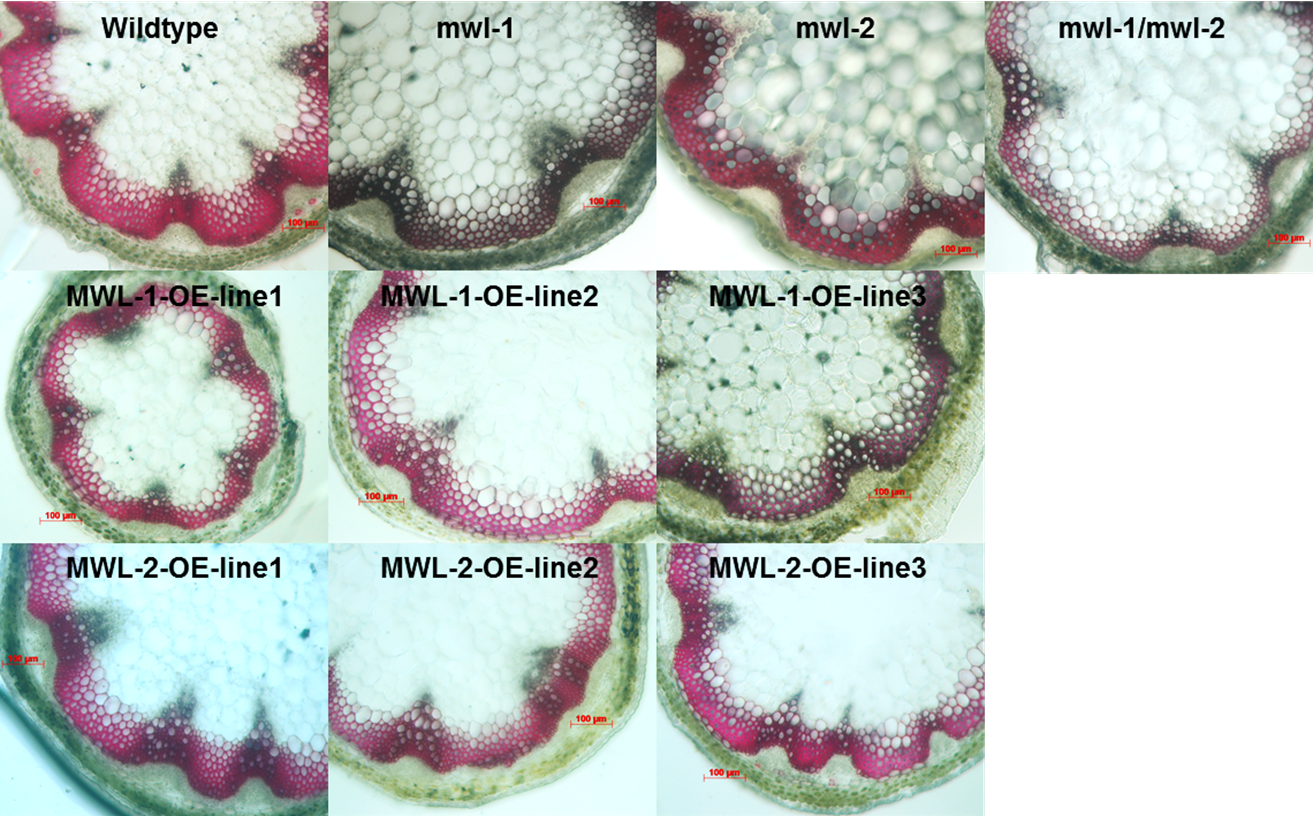


S5 Fig. Transverse sections of six-week-old stem tissue stained with phloroglucinol from At1g31720 (MWL-1) and At4g19370 (MWL-2) T-DNA knockout mutant and overexpression (OE) lines.

Transverse sections from wildtype (WT) (A), *At1g31720* mutant (B), *At4g19370* mutant (C), *At1g31720* x *At4g19370* double knockdown mutant (D), OEAt1g31720 line 1 (E), line 2 (F), line 3 (G), OEAt4g19370 line 1(H), line 2 (I), line 3 (J). Scale bar, 100μm (indicated in red). No discernible differences were seen in the transverse sections of the single and double mutant as well as the transgenic OE lines in comparison to the WT controls.
