# Supplementary material for: The Arabidopsis Domain of Unknown Function 1218 (DUF1218) Containing Proteins, MODIFYING WALL LIGNIN-1 and 2 (At1g31720/MWL-1 and At4g19370/MWL-2) Function Redundantly to Alter Secondary Cell Wall Lignin Content
Source: PLoS One. 2016 Mar 1;11(3):e0150254. doi: 10.1371/journal.pone.0150254 (PMC4773003; doi:10.1371/journal.pone.0150254)
Supplement: S1 Table — (DOCX) [file pone.0150254.s006.docx]

S1 Table. List of oligonucleotides used in the study.

| **Primer description** | **Primer sequence (5' to 3')** |
| --- | --- |
| SAIL screening primer (LB2) | GCTTCCTATTATATCTTCCCAAATTACCAATACA |
| GABI-Kat screening primer | TACTCATTGCTGATCCATGTAG |
| At1g31720 genomic screening primer | GCGTTCTTCCTATGCCTTTC |
| At4g19370 genomic screening primer | ACTTTGATCCTGGTGGCTGAA |
| At1g31720 transcript screening Fw | CCGTTGCGGTTACGTTGATA, |
| At1g31720 transcript screening Rv | TTCTCGACCTGTAATGATGG. |
| At4g19370 transcript screening Fw | CTTGGATCAGCCGCTGTTCT |
| At4g19370 transcript screening Rv | ACTTTGATCCTGGTGGCTGAA |
| Act2-Fw (At3g18780) | TGGAATCCACGAGACAACCT |
| Act2-Rv (At3g18780) | TGGACCTGCCTCATCATACT |
| OverExpressionAt1g31720-Fw | GCCATGGAAATTCAAAAACAAGATAA |
| OverExpressionAt1g31720-Rv | CTACACATTTTGAGTGTGACGTTTGTCAT |
| OverExpressionAt4g19370-Fw | GCCATGCACAACCTTTTTCTCTAC |
| OverExpressionAt4g19370-Rv | TTATATTCTGCTTATATTAGTGGAAGAC |
| 35S-specific primer | AGGAAGGTGGCTCCTA |
| Subcellular-At1g31720-Fw | GCCATGGAAATTCAAAAACAAGATAA |
| Subcellular-At1g31720-no stop-Rv | CACATTTTGAGTGTGACGTTTGTCATGAT |
| Subcellular-At4g19370-Fw | GCCATGCACAACCTTTTTCTCTAC |
| Subcellular-At4g19370-no stop-Rv | TATTCTGCTTATATTAGTGGAAGAC |
