# Supplementary material for: The Arabidopsis Domain of Unknown Function 1218 (DUF1218) Containing Proteins, MODIFYING WALL LIGNIN-1 and 2 (At1g31720/MWL-1 and At4g19370/MWL-2) Function Redundantly to Alter Secondary Cell Wall Lignin Content
Source: PLoS One. 2016 Mar 1;11(3):e0150254. doi: 10.1371/journal.pone.0150254 (PMC4773003; doi:10.1371/journal.pone.0150254)
Supplement: S2 Table — (DOCX) [file pone.0150254.s007.docx]

**S2 Table. Top 300 *Arabidopsis* co-expressed genes for MWL-1 (At1g31720) from ATTED-II represented as MR value.**

|  | **Locus** | **Alias** | **Function** | **MR value** |
| --- | --- | --- | --- | --- |
| 0 | At1g31720 | DUF1218 | Protein of unknown function (DUF1218) | 0 |
| 1 | At4g28500 | SND2 | NAC domain containing protein 73 | 5.5 |
| 2 | At1g27380 | RIC2 | ROP-interactive CRIB motif-containing protein 2 | 9.3 |
| 3 | At5g54690 | LGT6 | galacturonosyltransferase 12 | 13.6 |
| 4 | At2g41610 |  |  | 14.1 |
| 5 | At5g15630 | IRX6 | COBRA-like extracellular glycosyl-phosphatidyl inositol-anchored protein family | 15.6 |
| 6 | At5g16490 | RIC4 | ROP-interactive CRIB motif-containing protein 4 | 15.7 |
| 7 | At1g76250 |  |  | 19.3 |
| 8 | At3g50220 | IRX15 | Protein of unknown function (DUF579) | 20 |
| 9 | At4g18780 | LEW2 | cellulose synthase family protein | 20.3 |
| 10 | 840168 |  |  | 21.8 |
| 11 | At1g10460 | GLP7 | germin-like protein 7 | 22.8 |
| 12 | At1g58370 | RXF12 | glycosyl hydrolase family 10 protein / carbohydrate-binding domain-containing protein | 25.8 |
| 13 | At2g45040 | Matrixin | Matrixin family protein | 25.9 |
| *Table S2 cont.* | | | |  |
| 14 | At5g44030 | NWS2 | cellulose synthase A4 | 26.1 |
| 15 | At5g60720 | DUF547 | Protein of unknown function, DUF547 | 26.3 |
| 16 | At1g08340 | Rho GTPase activating with PAK-box/P21-Rho-binding domain | Rho GTPase activating protein with PAK-box/P21-Rho-binding domain | 27.1 |
| 17 | At3g18660 | PGSIP1 | plant glycogenin-like starch initiation protein 1 | 27.6 |
| 18 | At2g38060 | PHT4;2 | phosphate transporter 4;2 | 28.6 |
| 19 | At3g15050 | IQD10 | IQ-domain 10 | 29.4 |
| 20 | At5g14510 | ARM repeat | ARM repeat superfamily protein | 30.2 |
| 21 | At5g01360 | TBL3 | Plant protein of unknown function (DUF828) | 31.6 |
| 22 | At3g45870 | transporter | nodulin MtN21 /EamA-like transporter family protein | 31.8 |
| 23 | At5g60020 | LAC17 | laccase 17 | 31.9 |
| 24 | At1g09610 | DUF579 | Protein of unknown function (DUF579) | 32.2 |
| 25 | At5g17420 | MUR10 | Cellulose synthase family protein | 32.8 |
| 26 | At1g63910 | MYB103 | myb domain protein 103 | 33 |
| 27 | At3g08490 |  |  | 34.4 |
| 28 | At3g62020 | GLP10 | germin-like protein 10 | 34.5 |
| *Table S2 cont.* | | | | |
| 29 | At1g03920 | kinase | Protein kinase family protein | 34.8 |
| 30 | 832446 |  |  | 37.1 |
| 31 | At1g07120 |  |  | 38.1 |
| 32 | At4g27435 | DUF1218 | Protein of unknown function (DUF1218) | 38.9 |
| 33 | At1g27920 | MAP65-8 | microtubule-associated protein 65-8 | 39.6 |
| 34 | At5g45970 | ROP7 | RAC-like 2 | 39.7 |
| 35 | At2g37090 | IRX9 | Nucleotide-diphospho-sugar transferases superfamily protein | 40 |
| 36 | At1g29200 | transferase | O-fucosyltransferase family protein | 40.8 |
| 37 | At2g17940 | DUF827 | Plant protein of unknown function (DUF827) | 41.5 |
| 38 | At1g22480 | Cupredoxin | Cupredoxin superfamily protein | 42.5 |
| 39 | At2g27740 | DUF662 | Family of unknown function (DUF662) | 43.3 |
| 40 | At2g43840 | UGT74F1 | UDP-glycosyltransferase 74 F1 | 45.1 |
| 41 | At1g73640 | RABA6a | RAB GTPase homolog A6A | 46.5 |
| 42 | At1g28080 | RING finger | RING finger protein | 48.8 |
| 43 | At5g54570 | BGLU41 | beta glucosidase 41 | 49.5 |
| *Table S2 cont.* | | | | |
| 44 | At3g16920 | CTL2 | chitinase-like protein 2 | 49.9 |
| 45 | At5g47530 | Auxin-responsive | Auxin-responsive family protein | 50.2 |
| 46 | At4g33330 | PGSIP3 | plant glycogenin-like starch initiation protein 3 | 52 |
| 47 | At1g10800 |  |  | 54.2 |
| 48 | At2g38080 | LMCO4 | Laccase/Diphenol oxidase family protein | 54.3 |
| 49 | At1g54790 | hydrolase | GDSL-like Lipase/Acylhydrolase superfamily protein | 55 |
| 50 | At1g32100 | PRR1 | pinoresinol reductase 1 | 55.4 |
| 51 | At1g24030 | kinase | Protein kinase superfamily protein | 57.2 |
| 52 | At5g03170 | FLA11 | FASCICLIN-like arabinogalactan-protein 11 | 57.3 |
| 53 | At1g58070 |  |  | 57.4 |
| 54 | At2g15300 | kinase | Leucine-rich repeat protein kinase family protein | 58 |
| 55 | At1g23760 | PG3 | BURP domain-containing protein | 58.1 |
| 56 | At3g18670 | Ankyrin repeat | Ankyrin repeat family protein | 58.3 |
| 57 | At1g66810 | Zinc finger C-x8-C-x5-C-x3-H type | Zinc finger C-x8-C-x5-C-x3-H type family protein | 59.1 |
| 58 | At5g55950 | transporter | Nucleotide/sugar transporter family protein | 59.3 |
| *Table S2 cont.* | | | | |
| 59 | At4g17220 | MAP70-5 | microtubule-associated proteins 70-5 | 61.6 |
| 60 | At5g40630 | Ubiquitin-like | Ubiquitin-like superfamily protein | 62.5 |
| 61 | At2g29130 | LAC2 | laccase 2 | 63.5 |
| 62 | At5g01190 | LAC10 | laccase 10 | 63.6 |
| 63 | At5g05390 | LAC12 | laccase 12 | 63.6 |
| 64 | At4g18550 | DSEL | alpha/beta-Hydrolases superfamily protein | 66.4 |
| 65 | At5g12870 | MYB46 | myb domain protein 46 | 69.4 |
| 66 | At1g79420 | DUF620 | Protein of unknown function (DUF620) | 70.5 |
| 67 | At3g20450 | B-cell receptor-associated 31-like | B-cell receptor-associated protein 31-like | 70.7 |
| 68 | At2g37950 | RING | RING/FYVE/PHD zinc finger superfamily protein | 71 |
| 69 | At5g67210 | IRX15-L | Protein of unknown function (DUF579) | 71.2 |
| 70 | At1g63120 | RBL2 | RHOMBOID-like 2 | 71.5 |
| 71 | At5g11570 | Major facilitator | Major facilitator superfamily protein | 72.2 |
| 72 | At5g47635 | extensin | Pollen Ole e 1 allergen and extensin family protein | 72.8 |
| 73 | At4g22680 | MYB85 | myb domain protein 85 | 72.9 |
| *Table S2 cont.* | | | | |
| 74 | At1g17950 | MYB52 | myb domain protein 52 | 73.5 |
| 75 | At4g08160 | hydrolase | glycosyl hydrolase family 10 protein / carbohydrate-binding domain-containing protein | 73.5 |
| 76 | At3g62160 | transferase | HXXXD-type acyl-transferase family protein | 73.5 |
| 77 | At3g26125 | CYP86C2 | cytochrome P450, family 86, subfamily C, polypeptide 2 | 74.3 |
| 78 | At5g01930 | MAN6 | Glycosyl hydrolase superfamily protein | 76.1 |
| 79 | At4g21780 |  |  | 76.5 |
| 80 | At1g32770 | SND1 | NAC domain containing protein 12 | 77.1 |
| 81 | At4g16400 |  |  | 77.5 |
| 82 | At5g37180 | SUS5 | sucrose synthase 5 | 81.8 |
| 83 | At1g27440 | IRX10 | Exostosin family protein | 83.7 |
| 84 | At5g40020 | Pathogenesis-related thaumatin | Pathogenesis-related thaumatin superfamily protein | 84.6 |
| 85 | At1g05310 | Pectin lyase-like | Pectin lyase-like superfamily protein | 88 |
| 86 | At4g26140 | BGAL12 | beta-galactosidase 12 | 90.3 |
| 87 | At1g57670 | TIR | Toll-Interleukin-Resistance (TIR) domain family protein | 90.4 |
| 88 | At1g75720 | DUF827 | Plant protein of unknown function (DUF827) | 91 |
| *Table S2 cont.* | | | | |
| 89 | At3g56230 | BTB | BTB/POZ domain-containing protein | 92.2 |
| 90 | At1g63520 | DUF3527 | Protein of unknown function (DUF3527) | 92.3 |
| 91 | At5g11990 | proline-rich | proline-rich family protein | 94.3 |
| 92 | At1g68200 | Zinc finger C-x8-C-x5-C-x3-H type | Zinc finger C-x8-C-x5-C-x3-H type family protein | 94.4 |
| 93 | At1g47410 |  |  | 94.9 |
| 94 | At5g01070 | RING | RING/FYVE/PHD zinc finger superfamily protein | 97.5 |
| 95 | At3g08040 | MAN1 | MATE efflux family protein | 97.9 |
| 96 | At5g35960 | kinase | Protein kinase family protein | 99 |
| 97 | At5g22870 | LEA | Late embryogenesis abundant (LEA) hydroxyproline-rich glycoprotein family | 100.5 |
| 98 | At1g69080 | hydrolase | Adenine nucleotide alpha hydrolases-like superfamily protein | 100.6 |
| 99 | At5g38770 | GDU7 | glutamine dumper 7 | 101 |
| 100 | At1g67510 | kinase | Leucine-rich repeat protein kinase family protein | 101 |
| 101 | At4g33810 | hydrolase | Glycosyl hydrolase superfamily protein | 101.8 |
| 102 | At2g28650 | EXO70H8 | exocyst subunit exo70 family protein H8 | 101.8 |
| 103 | At1g28470 | SND3 | NAC domain containing protein 10 | 101.9 |
| *Table S2 cont.* | | | | |
| 104 | At1g43020 | DUF547 | Protein of unknown function, DUF547 | 103.2 |
| 105 | At1g08670 | ENTH | ENTH/VHS family protein | 103.5 |
| 106 | At5g03260 | LAC11 | laccase 11 | 105.2 |
| 107 | At4g24250 | MLO13 | Seven transmembrane MLO family protein | 105.3 |
| 108 | At1g79180 | MYB63 | myb domain protein 63 | 105.8 |
| 109 | At1g79620 | kinase | Leucine-rich repeat protein kinase family protein | 106.1 |
| 110 | At3g61910 | NST2 | NAC domain protein 66 | 107.5 |
| 111 | At5g04890 | RTM2 | HSP20-like chaperones superfamily protein | 109.9 |
| 112 | At1g09440 | kinase | Protein kinase superfamily protein | 111.5 |
| 113 | 824840 |  |  | 111.9 |
| 114 | At1g11915 |  |  | 112.4 |
| 115 | At3g27200 | Cupredoxin | Cupredoxin superfamily protein | 112.5 |
| 116 | At1g77700 | Pathogenesis-related thaumatin | Pathogenesis-related thaumatin superfamily protein | 113.5 |
| 117 | At2g46760 | oxidase | D-arabinono-1,4-lactone oxidase family protein | 114.4 |
| 118 | At5g38070 | RING | RING/FYVE/PHD zinc finger superfamily protein | 118.5 |
| *Table S2 cont.* | | | | |
| 119 | At1g31490 | transferase | HXXXD-type acyl-transferase family protein | 119.1 |
| 120 | At5g06200 | CASP4 | Uncharacterised protein family (UPF0497) | 119.3 |
| 121 | At4g14380 |  |  | 119.4 |
| 122 | At3g49070 | DUF677 | Protein of unknown function (DUF677) | 119.5 |
| 123 | At1g16490 | MYB58 | myb domain protein 58 | 119.8 |
| 124 | At2g31930 |  |  | 120.6 |
| 125 | At5g26330 | Cupredoxin | Cupredoxin superfamily protein | 122.8 |
| 126 | At4g05170 | DNA-binding | basic helix-loop-helix (bHLH) DNA-binding superfamily protein | 123.8 |
| 127 | At4g24430 | lyase | Rhamnogalacturonate lyase family protein | 124.7 |
| 128 | At5g48060 | transferase | C2 calcium/lipid-binding plant phosphoribosyltransferase family protein | 125 |
| 129 | At1g70500 | Pectin lyase-like | Pectin lyase-like superfamily protein | 126.4 |
| 130 | At4g02090 |  |  | 126.6 |
| 131 | At1g06490 | GSL7 | glucan synthase-like 7 | 126.8 |
| 132 | At5g03510 | zinc finger | C2H2-type zinc finger family protein | 127.1 |
| 133 | At2g38320 | TBL34 | TRICHOME BIREFRINGENCE-LIKE 34 | 127.8 |
| *Table S2 cont.* | | | | |
| 134 | At4g17905 | RING | RING/U-box superfamily protein | 127.8 |
| 135 | At5g26190 | Cysteine/Histidine-rich C1 domain | Cysteine/Histidine-rich C1 domain family protein | 128 |
| 136 | At5g45320 |  |  | 129 |
| 137 | At5g02640 |  |  | 129.5 |
| 138 | At1g71740 |  |  | 129.9 |
| 139 | At3g24110 | EF-hand | Calcium-binding EF-hand family protein | 131.4 |
| 140 | At5g48740 | kinase | Leucine-rich repeat protein kinase family protein | 132.2 |
| 141 | At5g67550 |  |  | 132.2 |
| 142 | At1g20850 | XCP2 | xylem cysteine peptidase 2 | 133.6 |
| 143 | At5g15290 | CASP5 | Uncharacterised protein family (UPF0497) | 134 |
| 144 | At5g67090 | endopeptidase | Subtilisin-like serine endopeptidase family protein | 134.3 |
| 145 | At5g38610 | inhibitor | Plant invertase/pectin methylesterase inhibitor superfamily protein | 136.1 |
| 146 | At1g55180 | PLDEPSILON | phospholipase D alpha 4 | 136.5 |
| 147 | At5g65530 | kinase | Protein kinase superfamily protein | 136.5 |
| 148 | At5g42180 | PER64 | Peroxidase superfamily protein | 138.3 |
| *Table S2 cont.* | | | | |
| 149 | At1g72220 | RING | RING/U-box superfamily protein | 140.9 |
| 150 | At5g46115 |  |  | 141.2 |
| 151 | At3g10340 | PAL4 | phenylalanine ammonia-lyase 4 | 141.6 |
| 152 | At3g49690 | RAX3 | myb domain protein 84 | 141.9 |
| 153 | At5g51890 | Peroxidase | Peroxidase superfamily protein | 142.8 |
| 154 | At4g35970 | APX5 | ascorbate peroxidase 5 | 144.7 |
| 155 | At1g72230 | Cupredoxin | Cupredoxin superfamily protein | 145.4 |
| 156 | At5g15900 | TBL19 | TRICHOME BIREFRINGENCE-LIKE 19 | 146.1 |
| 157 | At4g18510 | CLAVATA3 | CLAVATA3/ESR-related 2 | 146.5 |
| 158 | At2g44300 | inhibitor | Bifunctional inhibitor/lipid-transfer protein/seed storage 2S albumin superfamily protein | 148.5 |
| 159 | At5g07800 | monooxygenase | Flavin-binding monooxygenase family protein | 149 |
| 160 | At3g13590 | Cysteine/Histidine-rich C1 domain | Cysteine/Histidine-rich C1 domain family protein | 150.3 |
| 161 | At2g34540 |  |  | 150.8 |
| 162 | At5g60490 | FLA12 | FASCICLIN-like arabinogalactan-protein 12 | 151.8 |
| 163 | 827750 |  |  | 153.3 |
| *Table S2 cont.* | | | | |
| 164 | At5g49320 | DUF1218 | Protein of unknown function (DUF1218) | 157.9 |
| 165 | At4g28380 | LRR | Leucine-rich repeat (LRR) family protein | 158.4 |
| 166 | At3g59690 | IQD13 | IQ-domain 13 | 159.1 |
| 167 | At5g62380 | VND6 | NAC-domain protein 101 | 159.1 |
| 168 | At4g31330 | DUF599 | Protein of unknown function, DUF599 | 162 |
| 169 | At5g19870 | DUF716 | Family of unknown function (DUF716) | 162.5 |
| 170 | At1g61830 |  |  | 162.6 |
| 171 | At2g16980 | Major facilitator | Major facilitator superfamily protein | 162.8 |
| 172 | At5g24380 | YSL2 | YELLOW STRIPE like 2 | 164 |
| 173 | 821674 |  |  | 164 |
| 174 | At4g23496 | SP1L5 | SPIRAL1-like5 | 164.4 |
| 175 | At3g05620 | inhibitor | Plant invertase/pectin methylesterase inhibitor superfamily | 165.3 |
| 176 | At4g03340 | transferase | Core-2/I-branching beta-1,6-N-acetylglucosaminyltransferase family protein | 166.1 |
| 177 | At1g23530 |  |  | 167.3 |
| 178 | At5g56720 | c-NAD-MDH3 | Lactate/malate dehydrogenase family protein | 167.4 |
| *Table S2 cont.* | | | | |
| 179 | At3g58350 | RTM3 | RESTRICTED TEV MOVEMENT 3 | 169.6 |
| 180 | At2g41300 | SSL1 | strictosidine synthase-like 1 | 171.9 |
| 181 | At1g11570 | NTL | NTF2-like | 173.2 |
| 182 | At5g67230 | IRX14-L | Nucleotide-diphospho-sugar transferases superfamily protein | 173.9 |
| 183 | At1g29520 | AWPM-19-like | AWPM-19-like family protein | 174.1 |
| 184 | At1g66230 | MYB20 | myb domain protein 20 | 175.3 |
| 185 | At4g37445 |  |  | 175.8 |
| 186 | At5g02140 | Pathogenesis-related thaumatin | Pathogenesis-related thaumatin superfamily protein | 178.7 |
| 187 | At4g21310 | DUF1218 | Protein of unknown function (DUF1218) | 178.9 |
| 188 | At1g43790 | TED6 | tracheary element differentiation-related 6 | 179 |
| 189 | At3g47180 | RING | RING/U-box superfamily protein | 179.8 |
| 190 | 816888 |  |  | 180.2 |
| 191 | At3g26610 | Pectin lyase-like | Pectin lyase-like superfamily protein | 180.5 |
| 192 | At1g43650 | transporter | nodulin MtN21 /EamA-like transporter family protein | 182.3 |
| 193 | At1g64620 | DNA-binding | Dof-type zinc finger DNA-binding family protein | 182.3 |
|  |  |  |  |  |
| 194 | At1g63820 | CCT motif | CCT motif family protein | 182.6 |
| 195 | At4g35350 | XCP1 | xylem cysteine peptidase 1 | 184.6 |
| 196 | At1g05770 | Mannose-binding lectin | Mannose-binding lectin superfamily protein | 187.2 |
| 197 | At4g26320 | AGP13 | arabinogalactan protein 13 | 189.5 |
| 198 | At4g21340 | B70 | basic helix-loop-helix (bHLH) DNA-binding superfamily protein | 190.1 |
| 199 | At2g03200 | protease | Eukaryotic aspartyl protease family protein | 191.7 |
| 200 | At5g54980 | UPF0497 | Uncharacterised protein family (UPF0497) | 193.1 |
| 201 | At3g26350 |  |  | 193.3 |
| 202 | At2g39430 | dirigent-like | Disease resistance-responsive (dirigent-like protein) family protein | 193.5 |
| 203 | At1g59850 | ARM repeat | ARM repeat superfamily protein | 193.5 |
| 204 | At3g16340 | PDR1 | pleiotropic drug resistance 1 | 195.7 |
| 205 | At3g49820 |  |  | 196.4 |
| 206 | At2g28760 | UXS6 | UDP-XYL synthase 6 | 197.2 |
| 207 | At1g71930 | VND7 | vascular related NAC-domain protein 7 | 197.3 |
|  |  |  |  |  |
| *Table S2 cont.* | | | | |
| 208 | At1g05760 | RTM1 | Mannose-binding lectin superfamily protein | 199.3 |
| 209 | At5g18970 | AWPM-19-like | AWPM-19-like family protein | 199.5 |
| 210 | At3g59845 | dehydrogenase | Zinc-binding dehydrogenase family protein | 204.9 |
| 211 | At5g20870 | hydrolase | O-Glycosyl hydrolases family 17 protein | 205.5 |
| 212 | At2g22620 | lyase | Rhamnogalacturonate lyase family protein | 205.8 |
| 213 | At1g60030 | NAT7 | nucleobase-ascorbate transporter 7 | 206.5 |
| 214 | At2g47670 | inhibitor | Plant invertase/pectin methylesterase inhibitor superfamily protein | 206.7 |
| 215 | At4g00220 | LBD30 | Lateral organ boundaries (LOB) domain family protein | 207.1 |
| 216 | At2g04850 | Auxin-responsive | Auxin-responsive family protein | 207.5 |
| 217 | At1g31470 | NFD4 | Major facilitator superfamily protein | 208.6 |
| 218 | At2g44000 | LEA | Late embryogenesis abundant (LEA) hydroxyproline-rich glycoprotein family | 209.4 |
| 219 | At4g14650 |  |  | 209.7 |
| 220 | At3g11550 | CASP2 | Uncharacterised protein family (UPF0497) | 210.2 |
| 221 | 820874 |  |  | 210.7 |
| 222 | At3g18260 | Reticulon | Reticulon family protein | 213.1 |
| *Table S2 cont.* | | | | |
| 223 | 831872 |  |  | 214.1 |
| 224 | At5g19040 | IPT5 | isopentenyltransferase 5 | 214.6 |
| 225 | At3g10680 | chaperonin | HSP20-like chaperones superfamily protein | 216 |
| 226 | At1g18880 | Major facilitator | Major facilitator superfamily protein | 217.2 |
| 227 | At2g27370 | CASP3 | Uncharacterised protein family (UPF0497) | 218.4 |
| 228 | 834411 |  |  | 219 |
| 229 | At1g21140 | VIT | Vacuolar iron transporter (VIT) family protein | 219.8 |
| 230 | At1g04100 | IAA10 | indoleacetic acid-induced protein 10 | 223 |
| 231 | At2g18800 | XTH21 | xyloglucan endotransglucosylase/hydrolase 21 | 224.8 |
| 232 | At4g02810 | FAF1 | Protein of unknown function (DUF3049) | 227.1 |
| 233 | At5g51680 | glycoprotein | hydroxyproline-rich glycoprotein family protein | 227.4 |
| 234 | At5g53190 | SWEET3 | Nodulin MtN3 family protein | 228.9 |
| 235 | At5g61340 |  |  | 229.6 |
| 236 | At5g16600 | MYB43 | myb domain protein 43 | 231.3 |
| 237 | At5g01060 | Protein kinase with tetratricopeptide repeat domain | Protein kinase protein with tetratricopeptide repeat domain | 231.7 |
| *Table S2 cont.* | | | | |
| 238 | At2g40120 | kinase | Protein kinase superfamily protein | 234.1 |
| 239 | At2g19410 | kinase | U-box domain-containing protein kinase family protein | 236.5 |
| 240 | At5g61280 | Remorin | Remorin family protein | 239.1 |
| 241 | At1g12260 | VND4 | NAC 007 | 240.2 |
| 242 | At3g45700 | Major facilitator | Major facilitator superfamily protein | 242.4 |
| 243 | At3g15800 | hydrolase | Glycosyl hydrolase superfamily protein | 244.7 |
| 244 | At5g66815 |  |  | 245.4 |
| 245 | At4g15330 | CYP705A1 | cytochrome P450, family 705, subfamily A, polypeptide 1 | 246.4 |
| 246 | At1g61760 | LEA | Late embryogenesis abundant (LEA) hydroxyproline-rich glycoprotein family | 246.6 |
| 247 | At2g16970 | MEE15 | Major facilitator superfamily protein | 248.1 |
| 248 | At4g23900 | kinase | Nucleoside diphosphate kinase family protein | 248.6 |
| 249 | At1g19300 | PARVUS | Nucleotide-diphospho-sugar transferases superfamily protein | 248.7 |
| 250 | At1g47655 | DNA-binding | Dof-type zinc finger DNA-binding family protein | 249.6 |
| 251 | At5g40960 | Protein of unknown function | Protein of unknown function (DUF 3339) | 249.8 |
| 252 | At4g00230 | XSP1 | xylem serine peptidase 1 | 251 |
| *Table S2 cont.* | | | | |
| 253 | At2g38600 | HAD | HAD superfamily, subfamily IIIB acid phosphatase | 252.2 |
| 254 | At5g48690 |  |  | 252.5 |
| 255 | At1g19940 | GH9B5 | glycosyl hydrolase 9B5 | 252.9 |
| 256 | At1g50590 | RmlC-like cupins | RmlC-like cupins superfamily protein | 253.5 |
| 257 | 838626 |  |  | 253.8 |
| 258 | At1g72310 | RING | RING/U-box superfamily protein | 255 |
| 259 | At3g51710 | D-mannose binding lectin with Apple-like carbohydrate-binding domain | D-mannose binding lectin protein with Apple-like carbohydrate-binding domain | 260.6 |
| 260 | At1g05610 | APS2 | ADP-glucose pyrophosphorylase small subunit 2 | 261.2 |
| 261 | At1g27620 | transferase | HXXXD-type acyl-transferase family protein | 261.5 |
| 262 | At3g21550 | DMP2 | DUF679 domain membrane protein 2 | 262 |
| 263 | 831674 |  |  | 264.4 |
| 264 | At1g68230 | Reticulon | Reticulon family protein | 265.5 |
| 265 | At3g56700 | FAR6 | fatty acid reductase 6 | 266.8 |
| 266 | At3g17380 | TRAF-like | TRAF-like family protein | 267.5 |
| 267 | At4g22110 | dehydrogenase | GroES-like zinc-binding dehydrogenase family protein | 267.6 |
| *Table S2 cont.* | | | | |
| 268 | At3g52900 | DUF662 | Family of unknown function (DUF662) | 269.4 |
| 269 | At4g28330 |  |  | 269.9 |
| 270 | At1g78120 | TPR12 | Tetratricopeptide repeat (TPR)-like superfamily protein | 270.6 |
| 271 | At1g18140 | LAC1 | laccase 1 | 271.2 |
| 272 | At3g20830 | kinase | AGC (cAMP-dependent, cGMP-dependent and protein kinase C) kinase family protein | 271.6 |
| 273 | At2g31900 | XIF | myosin-like protein XIF | 277.4 |
| 274 | At5g61750 | RmlC-like cupins | RmlC-like cupins superfamily protein | 282.6 |
| 275 | At1g75090 | glycosylase | DNA glycosylase superfamily protein | 283.9 |
| 276 | At5g67050 | alpha/beta-Hydrolases | alpha/beta-Hydrolases superfamily protein | 285.9 |
| 277 | At5g62960 |  |  | 289.8 |
| 278 | At4g25434 | NUDT10 | nudix hydrolase homolog 10 | 290.2 |
| 279 | At2g22850 | bZIP6 | basic leucine-zipper 6 | 290.3 |
| 280 | At2g40160 | TBL30 | Plant protein of unknown function (DUF828) | 291.5 |
| 281 | At3g25855 | Copper transport | Copper transport protein family | 291.6 |
| 282 | At1g10380 | Putative membrane lipoprotein | Putative membrane lipoprotein | 292.8 |
| *Table S2 cont.* | | | | |
| 283 | At2g41200 |  |  | 293.8 |
| 284 | At5g11540 | oxidase | D-arabinono-1,4-lactone oxidase family protein | 294 |
| 285 | At2g36650 |  |  | 296 |
| 286 | At1g73370 | SUS6 | sucrose synthase 6 | 296.4 |
| 287 | 816231 |  |  | 300.1 |
| 288 | At1g31050 | DNA-binding | basic helix-loop-helix (bHLH) DNA-binding superfamily protein | 300.2 |
| 289 | At2g18380 | HANL1 | GATA transcription factor 20 | 303.1 |
| 290 | At1g62045 |  |  | 303.6 |
| 291 | At3g51300 | ROP1AT | RHO-related protein from plants 1 | 305 |
| 292 | At5g42690 | DUF547 | Protein of unknown function, DUF547 | 305.9 |
| 293 | At1g62990 | KNAT7 | KNOTTED-like homeobox of *Arabidopsis thaliana* 7 | 305.9 |
| 294 | At3g51030 | TRX1 | thioredoxin H-type 1 | 306 |
| 295 | At5g06850 | transferase | C2 calcium/lipid-binding plant phosphoribosyltransferase family protein | 306 |
| 296 | 835077 |  |  | 307.5 |
| 297 | 837811 |  |  | 309 |
| *Table S2 cont.* | | | | |
| 298 | At1g75280 | transcription | NmrA-like negative transcriptional regulator family protein | 309.2 |
| 299 | At1g13920 | Remorin | Remorin family protein | 309.2 |
| 300 | At1g71692 | XAL1 | AGAMOUS-like 12 | 310.8 |
