# Supplementary material for: The Arabidopsis Domain of Unknown Function 1218 (DUF1218) Containing Proteins, MODIFYING WALL LIGNIN-1 and 2 (At1g31720/MWL-1 and At4g19370/MWL-2) Function Redundantly to Alter Secondary Cell Wall Lignin Content
Source: PLoS One. 2016 Mar 1;11(3):e0150254. doi: 10.1371/journal.pone.0150254 (PMC4773003; doi:10.1371/journal.pone.0150254)
Supplement: S3 Table — (DOCX) [file pone.0150254.s008.docx]

**S3 Table. Top 300 *Arabidopsis* co-expressed genes for MWL-2 (At4g19370) from ATTED-II represented as MR value.**

|  | **Locus** | **Alias** | **Function** | **MR value** |
| --- | --- | --- | --- | --- |
| 0 | At4g19370 | DUF1218 | Protein of unknown function (DUF1218) | 0 |
| 1 | At5g02780 | GSTL1 | glutathione transferase lambda 1 | 2.8 |
| 2 | At1g51890 | kinase | Leucine-rich repeat protein kinase family protein | 5.6 |
| 3 | At5g61250 | GUS1 | glucuronidase 1 | 6.3 |
| 4 | At3g13950 |  |  | 9.9 |
| 5 | At5g38900 | Thioredoxin | Thioredoxin superfamily protein | 15.4 |
| 6 | At3g09020 | transferase | alpha 1,4-glycosyltransferase family protein | 17.6 |
| 7 | At1g33030 | transferase | O-methyltransferase family protein | 18.6 |
| 8 | At1g67980 | CCOAMT | caffeoyl-CoA 3-O-methyltransferase | 20.6 |
| 9 | At5g58540 | kinase | Protein kinase superfamily protein | 22.1 |
| 10 | At5g55560 | kinase | Protein kinase superfamily protein | 24.8 |
| 11 | At5g60280 | kinase | Concanavalin A-like lectin protein kinase family protein | 25.2 |
| 12 | At1g36640 |  |  | 25.3 |
| 13 | At1g74590 | GSTU10 | glutathione S-transferase TAU 10 | 25.5 |
| 14 | At1g67800 | Copine | Copine (Calcium-dependent phospholipid-binding protein) family | 25.7 |
| 15 | At1g01340 | CNGC10 | cyclic nucleotide gated channel 10 | 30.1 |
| 16 | At4g26120 | BTB/POZ | Ankyrin repeat family protein / BTB/POZ domain-containing protein | 30.3 |
| 17 | At1g26420 | Berberine | FAD-binding Berberine family protein | 30.9 |
| *Table S3 cont.* | | | | |
| 18 | At1g26380 | Berberine | FAD-binding Berberine family protein | 36.6 |
| 19 | At3g16030 | CES101 | lectin protein kinase family protein | 37.2 |
| 20 | At1g12290 | CC-NBS-LRR | Disease resistance protein (CC-NBS-LRR class) family | 38.5 |
| 21 | At5g59680 | kinase | Leucine-rich repeat protein kinase family protein | 40.2 |
| 22 | At3g18250 | Putative membrane lipoprotein | Putative membrane lipoprotein | 40.6 |
| 23 | At2g43000 | NAC042 | NAC domain containing protein 42 | 41.5 |
| 24 | At3g63380 | hydrolase | ATPase E1-E2 type family protein / haloacid dehalogenase-like hydrolase family protein | 41.8 |
| 25 | At4g38540 | FAD | FAD/NAD(P)-binding oxidoreductase family protein | 42.5 |
| 26 | At1g74360 | kinase | Leucine-rich repeat protein kinase family protein | 46.7 |
| 27 | At5g11210 | glutamate receptor 2.5 | glutamate receptor 2.5 | 47.7 |
| 28 | At5g64890 | PROPEP2 | elicitor peptide 2 precursor | 48.4 |
| 29 | At5g65600 | kinase | Concanavalin A-like lectin protein kinase family protein | 48.4 |
| 30 | At4g09110 | RING | RING/U-box superfamily protein | 49.5 |
| 31 | At1g62422 |  |  | 51.2 |
| 32 | At3g54150 | transferase | S-adenosyl-L-methionine-dependent methyltransferases superfamily protein | 52 |
| 33 | At4g29050 | kinase | Concanavalin A-like lectin protein kinase family protein | 53.2 |
| 34 | At1g49000 |  |  | 53.5 |
| 35 | At5g58940 | CRCK1 | calmodulin-binding receptor-like cytoplasmic kinase 1 | 58 |
| 36 | At1g79680 | WAKL10 | WALL ASSOCIATED KINASE (WAK)-LIKE 10 | 58.1 |
| *Table S3 cont.* | | | | |
| 37 | At5g08240 |  |  | 59.2 |
| 38 | At1g61560 | MLO6 | Seven transmembrane MLO family protein | 60.8 |
| 39 | At3g22460 | OASA2 | O-acetylserine (thiol) lyase (OAS-TL) isoform A2 | 62.2 |
| 40 | At1g51920 |  |  | 63.6 |
| 41 | At5g38210 | kinase | Protein kinase family protein | 63.6 |
| 42 | At4g31940 | CYP82C4 | cytochrome P450, family 82, subfamily C, polypeptide 4 | 64 |
| 43 | At5g45090 | PP2-A7 | phloem protein 2-A7 | 64.1 |
| 44 | At4g01010 | CNGC13 | cyclic nucleotide-gated channel 13 | 64.6 |
| 45 | At3g55470 | Calcium-dependent lipid-binding | Calcium-dependent lipid-binding (CaLB domain) family protein | 67.8 |
| 46 | At4g25110 | MCP1c | metacaspase 2 | 68.9 |
| 47 | At5g48410 | glutamate receptor 1.3 | glutamate receptor 1.3 | 69.8 |
| 48 | At5g16570 | GLN1;4 | glutamine synthetase 1;4 | 70.6 |
| 49 | At1g51270 | structural molecules;transmembrane receptors;structural molecules | structural molecules;transmembrane receptors;structural molecules | 72 |
| 50 | At3g09010 | kinase | Protein kinase superfamily protein | 72 |
| 51 | At1g65690 | LEA | Late embryogenesis abundant (LEA) hydroxyproline-rich glycoprotein family | 72.5 |
| 52 | At5g26920 | CBP60G | Cam-binding protein 60-like G | 72.8 |
| 53 | At4g39270 | kinase | Leucine-rich repeat protein kinase family protein | 75 |
| 54 | At1g18390 | kinase | Protein kinase superfamily protein | 75.7 |
| 55 | At4g21400 | CRK28 | cysteine-rich RLK (RECEPTOR-like protein kinase) 28 | 76.6 |
| *Table S3 cont.* | | | | |
| 56 | At4g16260 | hydrolase | Glycosyl hydrolase superfamily protein | 77 |
| 57 | At1g26410 | Berberine | FAD-binding Berberine family protein | 78.4 |
| 58 | At5g44990 | transferase | Glutathione S-transferase family protein | 81.3 |
| 59 | At5g11920 | cwINV6 | 6-&1-fructan exohydrolase | 82.8 |
| 60 | At3g09405 | Pectinacetylesterase | Pectinacetylesterase family protein | 83.4 |
| 61 | At5g58350 | ZIK2 | with no lysine (K) kinase 4 | 85 |
| 62 | At3g22260 | Cysteineases | Cysteine proteinases superfamily protein | 86.5 |
| 63 | At3g21780 | UGT71B6 | UDP-glucosyl transferase 71B6 | 86.5 |
| 64 | At4g31950 | CYP82C3 | cytochrome P450, family 82, subfamily C, polypeptide 3 | 87.3 |
| 65 | At2g30750 | CYP71A12 | cytochrome P450, family 71, subfamily A, polypeptide 12 | 87.9 |
| 66 | At3g22160 | VQ motif | VQ motif-containing protein | 87.9 |
| 67 | At3g53150 | UGT73D1 | UDP-glucosyl transferase 73D1 | 88.3 |
| 68 | At4g26060 | L18ae | Ribosomal protein L18ae family | 88.5 |
| 69 | At1g71400 | RLP12 | receptor like protein 12 | 88.5 |
| 70 | At5g53110 | RING | RING/U-box superfamily protein | 89 |
| 71 | At3g12900 | 2OG | 2-oxoglutarate (2OG) and Fe(II)-dependent oxygenase superfamily protein | 89.5 |
| 72 | At4g20110 | VSR7 | VACUOLAR SORTING RECEPTOR 7 | 89.5 |
| 73 | At5g10520 | RBK1 | ROP binding protein kinases 1 | 89.8 |
| 74 | At5g42440 | kinase | Protein kinase superfamily protein | 90 |
| *Table S3 cont.* | | | | |
| 75 | At3g01970 | WRKY45 | WRKY DNA-binding protein 45 | 90.8 |
| 76 | At3g14470 | NB-ARC disease resistance | NB-ARC domain-containing disease resistance protein | 90.9 |
| 77 | At4g23700 | CHX17 | cation/H+ exchanger 17 | 91.7 |
| 78 | At4g11850 | PLDGAMMA1 | phospholipase D gamma 1 | 91.8 |
| 79 | At2g23270 |  |  | 91.8 |
| 80 | At4g18250 | kinase | receptor serine/threonine kinase, putative | 91.9 |
| 81 | At3g26210 | CYP71B23 | cytochrome P450, family 71, subfamily B, polypeptide 23 | 92.7 |
| 82 | At5g48400 | Glutamate receptor | Glutamate receptor family protein | 92.9 |
| 83 | At1g52200 | PLAC8 | PLAC8 family protein | 93 |
| 84 | At1g23830 |  |  | 93.3 |
| 85 | 821503 |  |  | 94.5 |
| 86 | At4g23280 | CRK20 | cysteine-rich RLK (RECEPTOR-like protein kinase) 20 | 94.7 |
| 87 | At1g72540 | kinase | Protein kinase superfamily protein | 96.8 |
| 88 | At1g56160 | MYB72 | myb domain protein 72 | 100 |
| 89 | At2g38860 | YLS5 | Class I glutamine amidotransferase-like superfamily protein | 100.2 |
| 90 | At1g17330 | hydrolase | Metal-dependent phosphohydrolase | 100.7 |
| 91 | 840317 |  |  | 102 |
| 92 | At1g70690 | PDLP5 | Receptor-like protein kinase-related family protein | 102.9 |
| 93 | At1g18570 | MYB51 | myb domain protein 51 | 104.2 |
| *Table S3 cont.* | | | | |
| 94 | At2g02310 | PP2-B6 | phloem protein 2-B6 | 105.8 |
| 95 | At2g23830 | PapD-like | PapD-like superfamily protein | 106 |
| 96 | At1g64610 | Transducin/WD40 repeat-like | Transducin/WD40 repeat-like superfamily protein | 107.9 |
| 97 | At5g13080 | WRKY75 | WRKY DNA-binding protein 75 | 108.7 |
| 98 | At5g47960 | SMG1 | RAB GTPase homolog A4C | 109.9 |
| 99 | At5g62150 | LysM | peptidoglycan-binding LysM domain-containing protein | 110 |
| 100 | At1g13340 | Regulator of Vps4 activity in the MVB pathway | Regulator of Vps4 activity in the MVB pathway protein | 110 |
| 101 | At3g18560 |  |  | 111.7 |
| 102 | At1g10990 |  |  | 112.3 |
| 103 | At2g43140 | DNA-binding | basic helix-loop-helix (bHLH) DNA-binding superfamily protein | 114.6 |
| 104 | At1g57650 | ATP binding | ATP binding | 115.5 |
| 105 | At5g42830 | transferase | HXXXD-type acyl-transferase family protein | 116.3 |
| 106 | At5g07100 | WRKY26 | WRKY DNA-binding protein 26 | 117 |
| 107 | At2g23680 | WCOR413 | Cold acclimation protein WCOR413 family | 118.6 |
| 108 | At4g10510 | Subtilase | Subtilase family protein | 120.2 |
| 109 | At4g11170 | TIR-NBS-LRR | Disease resistance protein (TIR-NBS-LRR class) family | 120.5 |
| 110 | At1g09560 | GLP5 | germin-like protein 5 | 121.7 |
| 111 | At4g14630 | GLP9 | germin-like protein 9 | 125.2 |
| 112 | At4g18430 | RABA1e | RAB GTPase homolog A1E | 126.5 |
| *Table S3 cont.* | | | | |
| 113 | At5g18780 | F-box | F-box/RNI-like superfamily protein | 128.8 |
| 114 | At3g11840 | PUB24 | plant U-box 24 | 129.1 |
| 115 | At3g07600 | Heavy metal transport/detoxification | Heavy metal transport/detoxification superfamily protein | 129.5 |
| 116 | At1g28190 |  |  | 133.2 |
| 117 | At2g29990 | NDA2 | alternative NAD(P)H dehydrogenase 2 | 133.9 |
| 118 | At2g47130 | SDR3 | NAD(P)-binding Rossmann-fold superfamily protein | 134.5 |
| 119 | At5g39670 | EF-hand | Calcium-binding EF-hand family protein | 135.7 |
| 120 | At2g13810 | EDTS5 | AGD2-like defense response protein 1 | 136.5 |
| 121 | At5g20960 | AO1 | aldehyde oxidase 1 | 137 |
| 122 | At3g26440 | DUF707 | Protein of unknown function (DUF707) | 138.2 |
| 123 | 817758 |  |  | 138.3 |
| 124 | At3g26830 | PAD3 | Cytochrome P450 superfamily protein | 138.9 |
| 125 | At1g19250 | FMO1 | flavin-dependent monooxygenase 1 | 139.5 |
| 126 | At5g57220 | CYP81F2 | cytochrome P450, family 81, subfamily F, polypeptide 2 | 139.8 |
| 127 | At5g09290 | monophosphatase | Inositol monophosphatase family protein | 140.2 |
| 128 | At3g48850 | PHT3;2 | phosphate transporter 3;2 | 142.3 |
| 129 | At1g61420 | kinase | S-locus lectin protein kinase family protein | 142.4 |
| 130 | At1g56145 | kinase | Leucine-rich repeat transmembrane protein kinase | 142.5 |
| 131 | At3g11080 | RLP35 | receptor like protein 35 | 144.4 |
| *Table S3 cont.* | | | | |
| 132 | At5g07150 | kinase | Leucine-rich repeat protein kinase family protein | 144.9 |
| 133 | At2g35980 | YLS9 | Late embryogenesis abundant (LEA) hydroxyproline-rich glycoprotein family | 147.3 |
| 134 | At1g67810 | SUFE2 | sulfur E2 | 147.7 |
| 135 | At2g15390 | FUT4 | fucosyltransferase 4 | 148 |
| 136 | At2g19130 | kinase | S-locus lectin protein kinase family protein | 150.2 |
| 137 | At1g44130 | protease | Eukaryotic aspartyl protease family protein | 155.1 |
| 138 | At5g24230 | Lipase class 3 | Lipase class 3-related protein | 156.3 |
| 139 | At5g61010 | EXO70E2 | exocyst subunit exo70 family protein E2 | 156.3 |
| 140 | At5g64000 | SAL2 | Inositol monophosphatase family protein | 156.6 |
| 141 | At4g15270 | transferase | glucosyltransferase-related | 156.7 |
| 142 | At4g04960 | kinase | Concanavalin A-like lectin protein kinase family protein | 161.1 |
| 143 | At1g51790 | kinase | Leucine-rich repeat protein kinase family protein | 161.6 |
| 144 | At2g31230 | ERF15 | ethylene-responsive element binding factor 15 | 162.5 |
| 145 | At5g42010 | Transducin/WD40 repeat-like | Transducin/WD40 repeat-like superfamily protein | 163.9 |
| 146 | At2g26560 | PLP2 | phospholipase A 2A | 167.6 |
| 147 | At3g12040 | MAG | DNA-3-methyladenine glycosylase (MAG) | 167.6 |
| 148 | At1g47890 | RLP7 | receptor like protein 7 | 168.3 |
| 149 | At1g06160 | ORA59 | octadecanoid-responsive *Arabidopsis* AP2/ERF 59 | 168.3 |
| 150 | At5g67340 | ARM repeat | ARM repeat superfamily protein | 169.9 |
| *Table S3 cont.* | | | | |
| 151 | At3g52400 | SYP122 | syntaxin of plants 122 | 170.2 |
| 152 | At5g18490 | DUF946 | Plant protein of unknown function (DUF946) | 170.7 |
| 153 | At5g45000 | TIR-NBS-LRR | Disease resistance protein (TIR-NBS-LRR class) family | 172.8 |
| 154 | At5g62480 | GSTU9 | glutathione S-transferase tau 9 | 173.9 |
| 155 | At1g12940 | transporter | nitrate transporter2.5 | 174.1 |
| 156 | At5g64905 | PROPEP3 | elicitor peptide 3 precursor | 176.2 |
| 157 | At3g12820 | MYB10 | myb domain protein 10 | 177.2 |
| 158 | At1g03850 | GRXS13 | Glutaredoxin family protein | 177.4 |
| 159 | At5g25910 | RLP52 | receptor like protein 52 | 178.2 |
| 160 | At1g08940 | mutase | Phosphoglycerate mutase family protein | 179.7 |
| 161 | At2g19190 | FRK1 | FLG22-induced receptor-like kinase 1 | 179.9 |
| 162 | At4g23610 | LEA | Late embryogenesis abundant (LEA) hydroxyproline-rich glycoprotein family | 180 |
| 163 | At1g26390 | Berberine | FAD-binding Berberine family protein | 180.7 |
| 164 | At3g13610 | 2OG | 2-oxoglutarate (2OG) and Fe(II)-dependent oxygenase superfamily protein | 180.8 |
| 165 | At4g04490 | CRK36 | cysteine-rich RLK (RECEPTOR-like protein kinase) 36 | 183.5 |
| 166 | At3g59660 | GRAM | C2 domain-containing protein / GRAM domain-containing protein | 183.7 |
| 167 | At2g27660 | Cysteine/Histidine-rich C1 domain | Cysteine/Histidine-rich C1 domain family protein | 184.1 |
| 168 | At1g16420 | MCP2e | metacaspase 8 | 184.3 |
| 169 | At5g46780 | VQ motif | VQ motif-containing protein | 184.3 |
| *Table S3 cont.* | | | | |
| 170 | At1g22180 | Sec14p-like phosphatidylinositol transfer | Sec14p-like phosphatidylinositol transfer family protein | 184.9 |
| 171 | At2g04070 | MATE efflux | MATE efflux family protein | 185 |
| 172 | At1g02360 | Chitinase | Chitinase family protein | 187.2 |
| 173 | At1g05880 | RING | RING/U-box superfamily protein | 187.3 |
| 174 | At3g25610 | hydrolase | ATPase E1-E2 type family protein / haloacid dehalogenase-like hydrolase family protein | 187.9 |
| 175 | At3g49210 | WSD1-like | O-acyltransferase (WSD1-like) family protein | 188 |
| 176 | At1g32350 | AOX1D | alternative oxidase 1D | 189 |
| 177 | At1g69920 | GSTU12 | glutathione S-transferase TAU 12 | 189.3 |
| 178 | At5g45380 | DUR3 | solute:sodium symporters;urea transmembrane transporters | 191 |
| 179 | At5g27420 | CNI1 | carbon/nitrogen insensitive 1 | 191.3 |
| 180 | At4g01720 | WRKY47 | WRKY family transcription factor | 192.5 |
| 181 | At3g04720 | PR4 | pathogenesis-related 4 | 194.2 |
| 182 | At1g51860 | kinase | Leucine-rich repeat protein kinase family protein | 194.5 |
| 183 | At3g54420 | EP3 | homolog of carrot EP3-3 chitinase | 194.5 |
| 184 | At2g30770 | CYP71A13 | cytochrome P450, family 71, subfamily A, polypeptide 13 | 195.6 |
| 185 | At2g42360 | RING | RING/U-box superfamily protein | 196.4 |
| 186 | At1g07240 | UGT71C5 | UDP-glucosyl transferase 71C5 | 196.5 |
| 187 | At5g61160 | AACT1 | anthocyanin 5-aromatic acyltransferase 1 | 196.5 |
| 188 | At5g07390 | RBOHA | respiratory burst oxidase homolog A | 196.9 |
| *Table S3 cont.* | | | | |
| 189 | At3g51330 | protease | Eukaryotic aspartyl protease family protein | 199.3 |
| 190 | At2g18690 |  |  | 199.7 |
| 191 | At1g03740 | kinase | Protein kinase superfamily protein | 200 |
| 192 | At4g28460 |  |  | 200.1 |
| 193 | At4g33050 | EDA39 | calmodulin-binding family protein | 200.1 |
| 194 | At3g49370 | CDPK | Calcium-dependent protein kinase (CDPK) family protein | 202.6 |
| 195 | At1g10340 | Ankyrin repeat | Ankyrin repeat family protein | 202.7 |
| 196 | At1g57630 | TIR | Toll-Interleukin-Resistance (TIR) domain family protein | 203.3 |
| 197 | At3g59700 | LECRK1 | lectin-receptor kinase | 204 |
| 198 | At1g77660 | SET7/9 | Histone H3 K4-specific methyltransferase SET7/9 family protein | 206.8 |
| 199 | At1g14370 | PBL2 | protein kinase 2A | 207.8 |
| 200 | At2g43570 | CHI | chitinase, putative | 208.7 |
| 201 | At1g15790 |  |  | 209 |
| 202 | At4g37010 | CEN2 | centrin 2 | 209 |
| 203 | At1g61370 | kinase | S-locus lectin protein kinase family protein | 210.4 |
| 204 | At5g13320 | WIN3 | Auxin-responsive GH3 family protein | 211.1 |
| 205 | At3g46900 | COPT2 | copper transporter 2 | 213.9 |
| 206 | At4g38830 | CRK26 | cysteine-rich RLK (RECEPTOR-like protein kinase) 26 | 214.3 |
| 207 | At1g62200 | PTR6 | Major facilitator superfamily protein | 214.7 |
| *Table S3 cont.* | | | | |
| 208 | At5g45080 | PP2-A6 | phloem protein 2-A6 | 217.5 |
| 209 | At1g18860 | WRKY61 | WRKY DNA-binding protein 61 | 217.8 |
| 210 | At3g47480 | EF-hand | Calcium-binding EF-hand family protein | 217.8 |
| 211 | At5g35370 | kinase | S-locus lectin protein kinase family protein | 218.1 |
| 212 | At3g07720 | Galactose oxidase/kelch repeat | Galactose oxidase/kelch repeat superfamily protein | 219.1 |
| 213 | At4g23320 | CRK24 | cysteine-rich RLK (RECEPTOR-like protein kinase) 24 | 220.4 |
| 214 | At4g08780 | Peroxidase | Peroxidase superfamily protein | 220.7 |
| 215 | At3g56500 | serine-rich | serine-rich protein-related | 222.7 |
| 216 | At2g47190 | MYB2 | myb domain protein 2 | 224.6 |
| 217 | At5g62770 | DUF1645 | Protein of unknown function (DUF1645) | 225.6 |
| 218 | At1g21100 | IGMT1 | O-methyltransferase family protein | 225.8 |
| 219 | At4g39890 | RABH1c | RAB GTPase homolog H1C | 226.2 |
| 220 | At3g48640 |  |  | 226.4 |
| 221 | At5g46080 | kinase | Protein kinase superfamily protein | 227.3 |
| 222 | At5g22555 |  |  | 227.4 |
| 223 | At1g49050 | protease | Eukaryotic aspartyl protease family protein | 228.1 |
| 224 | At1g78410 | VQ motif | VQ motif-containing protein | 228.6 |
| 225 | At5g08335 | ICMTB | Isoprenylcysteine carboxyl methyltransferase (ICMT) family | 229.2 |
| 226 | At1g69450 | ERD4 | Early-responsive to dehydration stress protein (ERD4) | 230.4 |
| *Table S3 cont.* | | | | |
| 227 | At1g63720 |  |  | 230.7 |
| 228 | At1g14070 | FUT7 | fucosyltransferase 7 | 231.6 |
| 229 | At3g61390 | RING | RING/U-box superfamily protein | 231.6 |
| 230 | At3g47050 | hydrolase | Glycosyl hydrolase family protein | 233.1 |
| 231 | At5g15130 | WRKY72 | WRKY DNA-binding protein 72 | 233.4 |
| 232 | At2g42060 | Cysteine/Histidine-rich C1 domain | Cysteine/Histidine-rich C1 domain family protein | 234 |
| 233 | At5g67310 | CYP81G1 | cytochrome P450, family 81, subfamily G, polypeptide 1 | 234.2 |
| 234 | At4g31875 |  |  | 234.3 |
| 235 | At4g18990 | XTH29 | xyloglucan endotransglucosylase/hydrolase 29 | 236.3 |
| 236 | At3g48890 | MSBP2 | membrane-associated progesterone binding protein 3 | 237.1 |
| 237 | At1g05700 | kinase | Leucine-rich repeat transmembrane protein kinase protein | 239.3 |
| 238 | At1g53990 | GLIP3 | GDSL-motif lipase 3 | 240.2 |
| 239 | At3g21080 | transporter | ABC transporter-related | 240.6 |
| 240 | At4g38260 | DUF833 | Protein of unknown function (DUF833) | 241 |
| 241 | At2g29110 | glutamate receptor 2.8 | glutamate receptor 2.8 | 243.5 |
| 242 | At1g23840 |  |  | 245 |
| 243 | At3g55910 |  |  | 246.2 |
| 244 | At1g61360 | kinase | S-locus lectin protein kinase family protein | 247.7 |
| 245 | At1g16150 | WAKL4 | wall associated kinase-like 4 | 248.9 |
| *Table S3 cont.* | | | | |
| 246 | At4g08770 | Prx37 | Peroxidase superfamily protein | 249.4 |
| 247 | At3g23550 | MATE efflux | MATE efflux family protein | 249.8 |
| 248 | At5g40990 | GLIP1 | GDSL lipase 1 | 250 |
| 249 | At3g21520 | DMP1 | DUF679 domain membrane protein 1 | 250.4 |
| 250 | At1g21240 | WAK3 | wall associated kinase 3 | 250.4 |
| 251 | At3g52430 | PAD4 | alpha/beta-Hydrolases superfamily protein | 252.5 |
| 252 | At1g68450 | PDE337 | VQ motif-containing protein | 253.3 |
| 253 | At4g20000 | VQ motif | VQ motif-containing protein | 253.7 |
| 254 | At1g02170 | MCP1b | metacaspase 1 | 253.9 |
| 255 | At2g42350 | RING | RING/U-box superfamily protein | 255.6 |
| 256 | At5g20400 | 2OG | 2-oxoglutarate (2OG) and Fe(II)-dependent oxygenase superfamily protein | 255.8 |
| 257 | At2g20900 | DGK5 | diacylglycerol kinase 5 | 256 |
| 258 | At3g62150 | PGP21 | P-glycoprotein 21 | 256.4 |
| 259 | At3g13100 | MRP7 | multidrug resistance-associated protein 7 | 256.4 |
| 260 | At3g56410 | DUF3133 | Protein of unknown function (DUF3133) | 257.6 |
| 261 | At5g18310 |  |  | 257.8 |
| 262 | At2g30840 | 2OG | 2-oxoglutarate (2OG) and Fe(II)-dependent oxygenase superfamily protein | 258 |
| 263 | At3g45330 | kinase | Concanavalin A-like lectin protein kinase family protein | 258.5 |
| 264 | At2g45220 | inhibitor | Plant invertase/pectin methylesterase inhibitor superfamily | 258.8 |
| *Table S3 cont.* | | | | |
| 265 | At3g15510 | NARS1 | NAC domain containing protein 2 | 259.8 |
| 266 | At4g30230 |  |  | 260.3 |
| 267 | At3g60120 | BGLU27 | beta glucosidase 27 | 261.1 |
| 268 | 829620 |  |  | 261.3 |
| 269 | At1g59590 | ZCF37 | ZCF37 | 261.7 |
| 270 | At2g44010 |  |  | 262.2 |
| 271 | At4g11650 | OSM34 | osmotin 34 | 262.6 |
| 272 | At4g21120 | CAT1 | amino acid transporter 1 | 263.2 |
| 273 | At1g23850 |  |  | 263.4 |
| 274 | At1g34420 | kinase | leucine-rich repeat transmembrane protein kinase family protein | 263.5 |
| 275 | At3g46690 | transferase | UDP-Glycosyltransferase superfamily protein | 263.7 |
| 276 | At5g05300 |  |  | 264.9 |
| 277 | At2g04430 | NUDT5 | nudix hydrolase homolog 5 | 265 |
| 278 | At1g66880 | kinase | Protein kinase superfamily protein | 265.6 |
| 279 | At1g16670 | kinase | Protein kinase superfamily protein | 266.6 |
| 280 | At3g12740 | ALIS1 | ALA-interacting subunit 1 | 266.9 |
| 281 | At5g12930 |  |  | 268.9 |
| 282 | At3g50740 | UGT72E1 | UDP-glucosyl transferase 72E1 | 268.9 |
| 283 | 825366 |  |  | 269.2 |
| *Table S3 cont.* | | | | |
| 284 | At1g07000 | EXO70B2 | exocyst subunit exo70 family protein B2 | 269.4 |
| 285 | 836574 |  |  | 270.8 |
| 286 | At1g70920 | HB18 | homeobox-leucine zipper protein 18 | 272.7 |
| 287 | At1g74080 | MYB122 | myb domain protein 122 | 273.2 |
| 288 | At4g00700 | transferase | C2 calcium/lipid-binding plant phosphoribosyltransferase family protein | 273.3 |
| 289 | At1g10140 | UCP031279 | Uncharacterised conserved protein UCP031279 | 274.2 |
| 290 | At2g28710 | zinc finger | C2H2-type zinc finger family protein | 274.7 |
| 291 | At3g50770 | CML41 | calmodulin-like 41 | 275.8 |
| 292 | At5g37740 | Calcium-dependent lipid-binding | Calcium-dependent lipid-binding (CaLB domain) family protein | 275.9 |
| 293 | At3g28580 | hydrolase | P-loop containing nucleoside triphosphate hydrolases superfamily protein | 276.6 |
| 294 | At3g55890 | Yippee putative zinc-binding | Yippee family putative zinc-binding protein | 277 |
| 295 | At5g54140 | ILL3 | IAA-leucine-resistant (ILR1)-like 3 | 277.4 |
| 296 | 828696 |  |  | 278.5 |
| 297 | At1g76970 | Target of Myb 1 | Target of Myb protein 1 | 278.5 |
| 298 | At3g09490 | Tetratricopeptide repeat (TPR)-like | Tetratricopeptide repeat (TPR)-like superfamily protein | 278.8 |
| 299 | At5g37070 | DUF538 | Protein of unknown function, DUF538 | 279.3 |
| 300 | At1g24140 | Matrixin | Matrixin family protein | 279.4 |
