# Supplementary material for: The Arabidopsis Domain of Unknown Function 1218 (DUF1218) Containing Proteins, MODIFYING WALL LIGNIN-1 and 2 (At1g31720/MWL-1 and At4g19370/MWL-2) Function Redundantly to Alter Secondary Cell Wall Lignin Content
Source: PLoS One. 2016 Mar 1;11(3):e0150254. doi: 10.1371/journal.pone.0150254 (PMC4773003; doi:10.1371/journal.pone.0150254)
Supplement: S4 Table — (DOCX) [file pone.0150254.s009.docx]

S4 Table. Cell wall lignin content from MWL-1 and MWL-2 overexpression, single and double knockout lines compared to its respective wildtype (WT) control.

|  | COMPONENTS (% of wood) | | |
| --- | --- | --- | --- |
|  | Lignin (% of dry mass) | | |
| **Sample** | **Soluble lignin (%)** | **Insoluble lignin (%)** | **Total lignin (%)** |
| MWL-2-WT | 5.05 ±0.36 | 15.13 ±0.57 | 20.18 ±0.62 |
| mwl-2 | 5.46 ±0.27 | 15.69 ±0.89 | 21.15 ±0.66 |
| *P*-value | 0.07 | 0.59 | 0.41 |
| Percentage fold-change | - | - | - |
| Change relative to WT | - | - | - |
|  |  |  |  |
| MWL-1-WT | 3.72 ±0.26 | 18.44 ±0.25 | 22.16 ±0.28 |
| mwl-1 | 3.85 ±0.29 | 18.58 ±0.09 | 22.43 ±0.27 |
| *P*-value | 0.5 | 0.65 | 0.32 |
| Percentage fold-change | - | - | - |
| Change relative to WT | - | - | - |
|  |  |  |  |
| WT | 2.5 ±0.04 | 18.45 ±0.20 | 20.95 ±0.20 |
| mwl-1/mwl-2 | 2.95 ±0.03 | 15.97 ±0.24 | 18.92 ±0.27 |
| P-value | **0.02** | **0.01** | **0.02** |
| Percentage fold-change | **18** | **15.53** | **10.73** |
| Change relative to WT | **Increase** | **Decrease** | **Decrease** |
|  |  |  |  |
| OE-MWL-1-line1-WT | 4.8 ±0.17 | 18.4 ±0.38 | 23.2 ±0.22 |
| OE-MWL-1 | 4.9 ±0.27 | 17.29 ±0.35 | 22.18 ±0.14 |
| *P*-value | 0.44 | 0.003 | 0.02 |
| Percentage fold-change | - | 6.420 | 4.60 |
| Change relative to WT | - | **Decrease** | **Decrease** |
|  |  |  |  |
| OE-MWL-1-line2-WT | 4.47 ±0.22 | 17.5 ±0.33 | 21.97 ±0.11 |
| OE-MWL-1-line2 | 3.93 ±0.00 | 18.69 ±0.03 | 22.62 ±0.03 |
| *P*-value | 0.25 | 0.19 | 0.07 |
| Percentage fold-change | - | - | - |
| Change relative to WT | - | - | - |
|  |  |  |  |
|  |  |  |  |
|  |  |  |  |
| *Table S4 cont*. |  |  |  |
| OE-MWL-1-line3-WT | 4.09 ±0.17 | 18.5 ±0.55 | 22.59 ±0.38 |
| OE-MWL-1-line3 | 4.21 ±0.03 | 19.13 ±0.15 | 23.33 ±0.12 |
| *P*-value | 0.22 | 0.79 | 0.14 |
| Percentage fold-change | - | - | - |
| Change relative to WT | - | - | - |
|  |  |  |  |
| OE-MWL-2-line1-WT | 4.69 ±0.30 | 19.86 ±0.88 | 24.54 ±1.18 |
| OE-MWL-2-line1 | 4.2 ±0.08 | 19.2 ±1.28 | 23.4 ±1.36 |
| *P*-value | 0.42 | 0.81 | 0.73 |
| Percentage fold-change | - | - | - |
| Change relative to WT | - | - | - |
|  |  |  |  |
| OE-MWL-2-line2-WT | 4.5 ±0.22 | 18.18 ±0.21 | 22.68 ±0.18 |
| OE-MWL-2-line2 | 4.49 ±0.18 | 16.58 ±0.26 | 21.08 ±0.23 |
| *P*-value | 0.92 | 0.04 | 0.06 |
| Percentage fold-change | - | 9.65 | - |
| Change relative to WT | - | **Decrease** | - |
|  |  |  |  |
| OE-MWL-2-line3-WT | 4.16 ±0.13 | 20.86 ±0.07 | 25.02 ±0.20 |
| OE-MWL-2-line3 | 4.38 ±0.54 | 15.89 ±0.68 | 20.27 ±0.14 |
| *P*-value | 0.68 | 0.1 | **0.05** |
| Percentage fold-change | - | - | 23.43 |
| Change relative to WT | - | - | **Decrease** |

Values represent the mean of three biological repeats (containing bulked tissue of ~24 plants each) plus/minus the standard error of mean, while significance was calculated using a two-tailed Student’s *t*-tests with α of 0.05 as significance threshold.
